# Effectiveness and Tolerability of Different Recommended Doses of PPIs and H_2_RAs in GERD: Network Meta-Analysis and GRADE system

**DOI:** 10.1038/srep41021

**Published:** 2017-01-19

**Authors:** Chao Zhang, Joey S. W. Kwong, Rui-Xia Yuan, Hao Chen, Chang Xu, Yi-Pin Wang, Gong-Li Yang, Jin-Zhu Yan, Le Peng, Xian-Tao Zeng, Hong Weng, Jie Luo, Yu-Ming Niu

**Affiliations:** 1 Center for Evidence-Based Medicine and Clinical Research, Taihe Hospital, Hubei University of Medicine, Shiyan 442000, Hubei Province, China; 2 Chinese Cochrane Center, Chinese Evidence-Based Medicine Center, West China Hospital, Sichuan University, Chengdu 610041, Sichuan Province, China; 3 Department of Epidemiology and Biostatistics, School of Public Health, Wuhan University and Global Health Institute, Wuhan University, Wuhan 430000, Hubei Province, China; 4 Center for Evidence-Based Medicine, Nanjing University of Chinese Medicine, Nanjing 210000, Jiangsu Province, China; 5 Department of Urology, Zhongnan Hospital of Wuhan University, Wuhan University, Wuhan 430000, Hubei Province, China; 6 Department of Gastroenterology, Taihe Hospital, Hubei University of Medicine, Shiyan 442000, Hubei Province, China

## Abstract

Proton pump inhibitors (PPIs) and histamine-2-receptor antagonists (H_2_RAs) are used for gastro-esophageal reflux disease (GERD); however, the clinical evidence for treatment is poor. We evaluated the effectiveness and tolerability of different doses of PPIs, H_2_RAs and placebo in adults with GERD. Six online databases were searched through September 1, 2016. All related articles were included and combined with a Bayesian network meta-analysis from randomized controlled trials (RCTs). The GRADE systems were employed to assess the main outcome. Ninety-eight RCTs were identified, which included 45,964 participants. Our analysis indicated that the full/standard dose of esomeprazole at 40 mg per day was the most efficient in healing among nine different dosages of PPIs and H_2_RAs. The main efficacy outcome did not change after adjustments for the area, age, level of disease from endoscopy, year of publication, pharmaceutical industry sponsorship, Intention-to-treat (ITT)/per-protocol (PP), withdrawal rate, pre-set select design bias, single blinded and unblinded studies, study origination in China, study arms that included zero events, inconsistency node or discontinued drug were accounted for in the meta-regressions and sensitivity analyses. This research suggests that the full/standard doses (40 mg per day) of esomeprazole should be recommended as first-line treatments for GERD in adults for short-term therapy.

Gastro-esophageal reflux disease (GERD) reflects symptoms or mucosal damage caused by the reflux of gastric contents from the stomach into the esophagus^1^. It affects approximately 20–30% of the population worldwide and is particularly evident in Western countries^2^.

GERD is typically caused by changes in the barrier between the stomach and the esophagus, including abnormal relaxation of the lower esophageal sphincter, which typically holds the top of the stomach closed, impaired expulsion of gastric reflux from the esophagus, or a hiatal hernia. The corresponding GERD symptoms include heartburn, regurgitation, odynophagia, nausea, chest pain and coughing^3^. Without effective treatment, complications worsen and further develop into reflux esophagitis, esophageal strictures, and Barrett’s esophagus^3^,^4^,^5^, and in severe cases, esophageal adenocarcinoma may also occur^6^,^7^.

Currently, the main treatment options for GERD include drug therapy, surgery, and lifestyle changes^8^,^9^,^10^,^11^,^12^. The most important and widely used therapeutic regimen is drug therapy, which includes treatment with proton pump inhibitors (PPIs) and histamine-2-receptor antagonists (H_2_RAs)^9^,^11^,^13^. However, the use of pharmacological agents comes with the concern of tolerability^14^,^15^,^16^, defined as discontinuation caused for any reason, including ineffectiveness, adverse effects and a lack of compliance. There is substantial evidence for the effectiveness and tolerability of pharmacological agents in the treatment of GERD^14^,^17^,^18^,^19^,^20^,^21^. However, most of these findings have been obtained from pairwise comparisons within each class of drugs. A previous network meta-analysis of 27 randomized studies has indicated that PPIs were more effective as anti-reflux agents than H_2_RAs in terms of healing^22^. However, no information was available for each individual drug, and the types of outcome measures were limited. Consequently, our efforts to obtain accurate and up-to-date information regarding the properties of pharmacological interventions for GERD led us to pursue a Bayesian network meta-analysis, which combined both direct and indirect evidence for multiple treatment comparisons; these findings would inform us of the clinical efficacy and tolerability of both medication classes used in the short-term treatment of GERD.

## Results

### Characteristics of eligible trials

**Figure 1:**
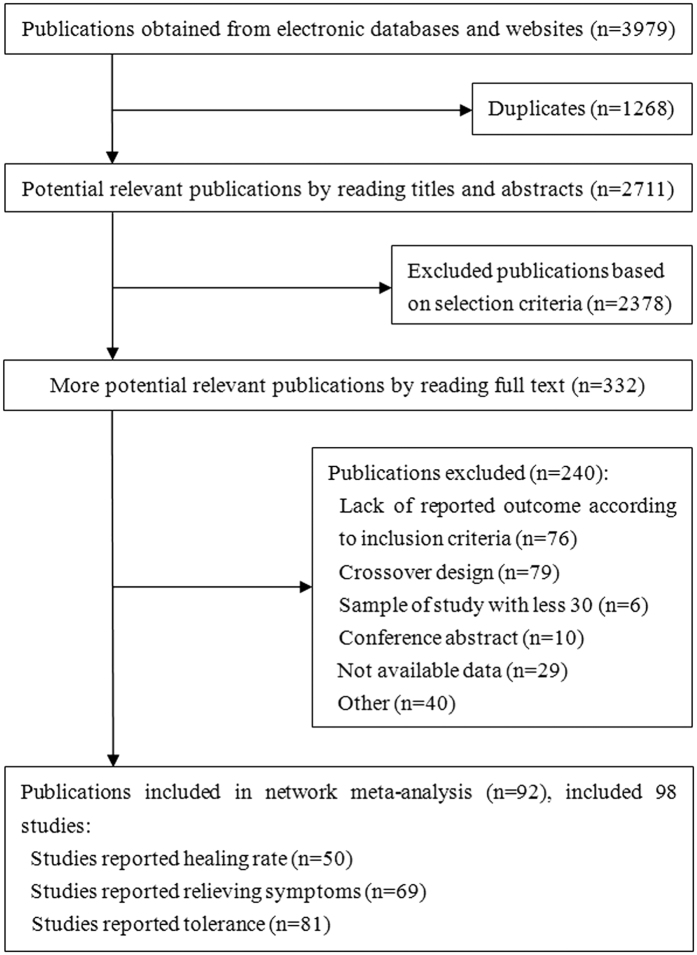
Summary of trial identification and selection.

Our systematic literature search identified 3,979 potential publications (Fig. 1). Based on the selection criteria, we obtained quantitative data for our network meta-analysis by reading all titles, abstracts, and full text evaluations. We ultimately included 98 randomized controlled trials with 45,964 enrolled participants, which included 40,927 participants who received interventions and 5,037 participants who received placebos. Nine interventions were used, including five PPIs (esomeprazole, lansoprazole, pantoprazole, omeprazole, and rabeprazole) and four H_2_RAs (cimetidine, famotidine, nizatidine, and ranitidine).

**Figure 2:**
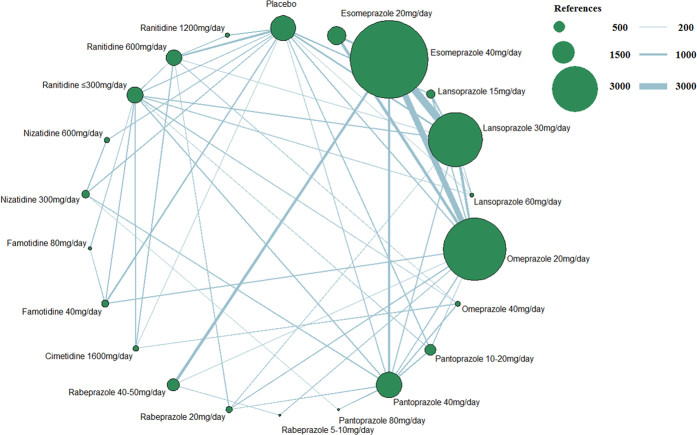
Network figure for healing. (The node sizes correspond to the number of trials that investigated the treatments. Directly comparable treatments are linked with a line, and the thickness of the line corresponds to the sample size in each pairwise treatment comparison. The “References” at the upper right corner displays three different nodes sizes correspond to three different levels of sample size of placebo and active drugs, three different lines thickness correspond to the three levels of different sample size of each pairwise treatment comparison).

**Figure 3:**
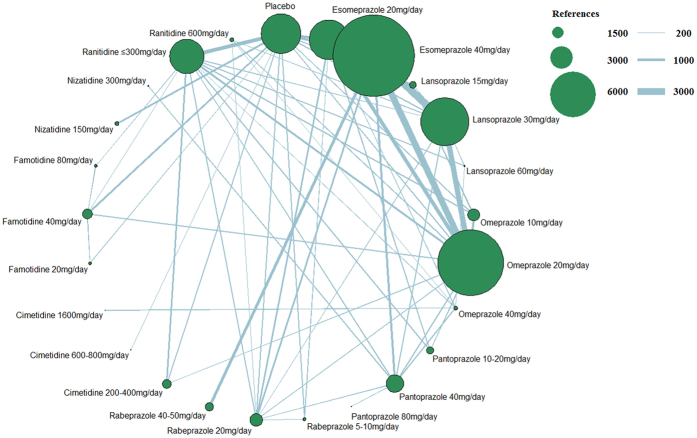
Network figure for relief of symptoms. (The node sizes correspond to the number of trials that investigated the treatments. Directly comparable treatments are linked with a line, and the thickness of the line corresponds to the sample size in each pairwise treatment comparison. The “References” at the upper right corner displays three different nodes sizes correspond to three different levels of sample size of placebo and active drugs, three different lines thickness correspond to the three levels of different sample size of each pairwise treatment comparison).

**Figure 4:**
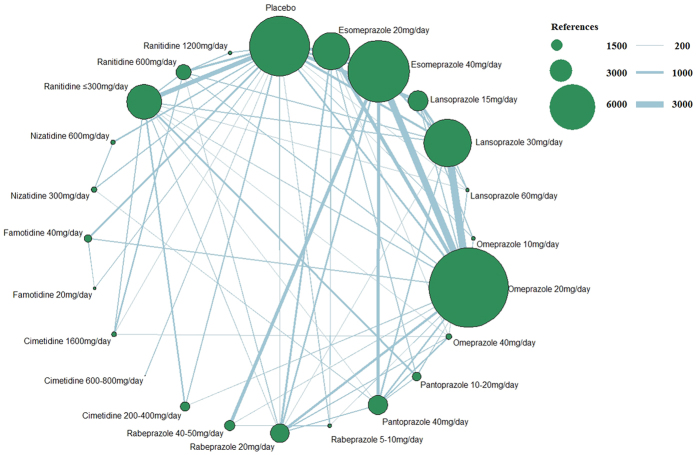
Network figure for tolerance. (The node sizes correspond to the number of trials that investigated the treatments. Directly comparable treatments are linked with a line, and the thickness of the line corresponds to the sample size in each pairwise treatment comparison. The “References” at the upper right corner displays three different nodes sizes correspond to three different levels of sample size of placebo and active drugs, three different lines thickness correspond to the three levels of different sample size of each pairwise treatment comparison).

Figure 2 presents the network of eligible studies and dose comparisons for the main outcomes, Fig. 3 indicates the relief of symptoms, and Fig. 4 presents the tolerance. Healing was reported in 50 studies (22,669 of 29,392 participants), with 12 studies including placebo; relief of symptoms was reported in 69 studies (41,373 participants), with 22 studies including placebo; and data on drug tolerance were available in 81 studies (42,341 participants), with 31 studies including placebo.

### Characteristics of methodological quality and industry sponsored bias

The overall methodological quality of the included studies was good, and the overall mean score was 4, based on the methodological attributes. The study score distribution range was 1 to 6 points: 1 trial had one point, 9 trials had two points, 36 trials had three points, 21 trials had four points, and 16 trials had five points. Only 15 trials were rated as having high quality for all six methodological quality attributes. Five trials were not double-blind, 2 trials were single-blind, and 31 trials were double-blind; the methodological quality of the other studies were not clear. The methodological quality of the included trials is presented in the summary plot for the risk of bias (Supplementary Fig. 1a–d, respectively).

Of 98 trials, 64 trials exhibited industry-sponsored bias, and all drugs involved it. We determined the distribution of the corresponding numbers of trials for sponsored drugs: 19 for esomeprazole, 8 for lansoprazole, 25 for omeprazole, 14 for pantoprazole, 9 for rabeprazole, 3 for cimetidine, 2 for famotidine, 2 for nizatidine, and 15 for ranitidine. The number of PPIs (77.6%) was relatively greater than H_2_RAs (22.4%) (Supplementary Table S1).

### Potential confounding factors, design bias, and consistency

We conducted meta-regression analyses to evaluate the baseline, influence of confounding factors on the results and heterogeneity in a consistency regression model. The majority of confounding factors did not reach statistical significance in all outcomes (Supplementary Table S2), whereas gender (B = −0.127 (−0.225, −0.026), P = 0.010) and overall methodological quality score (B = −1.006 (−1.808, −0.213), P = 0.012) were significant for the main outcome; the year of publication for the tolerance (B = 0.028 (0.008, 0.049), P = 0.007) was also significant in consistent coefficients model.

Based on pre-set selective design biases for various scenarios, we comprehensively evaluated all results (Supplementary Table S3). For the healing and tolerance outcomes, our results did not provide sufficient evidence to support design bias (new favored bias, sponsored favored bias, high-dose favored bias, and PPI favored bias), regardless of whether the supplementary merger favored bias. For relief of symptoms, the PPIs favored a design bias based on the finding that the fixed and exchangeable coefficients reached statistical significance (B_f_ = 4.49 (1.26, 7.85), P = 0.007; and B_e_ = 2.29 (1.32, 7.55), P = 0.045). The supplementary merger PPI favored and sponsored favored bias subsequently reached statistical significance. Based on these adjusted results and the existing clinical advantages of PPIs and high-dose interventions, the DIC of the hierarchical modeling approach that incorporated dose-related and PPI favored constraints and the previously described factors exhibited more obvious advantages compared with the other models (Supplementary Table S4).

Based on the “node-splitting” method, we determined that most nodes from all outcomes had superior consistency for all specific comparisons (node) with “direct” and “indirect” evidence apart from the relief of symptoms outcome. Based on the “node-splitting” method to separate potential confounding factors, we determined that this poor state for relief of symptoms was improved after separating out pharmaceutical industry sponsorship. However, when excluding the key drug with inconsistent nodes (lansoprazole at 15 mg per day), the situation was not reversed. The causes and management of inconsistencies will be described in our discussion. For the main and tolerance outcomes, individual inconsistency nodes were identified and subsequently removed in the sensitivity analysis. Finally, the inconsistency model demonstrated no clear advantage.

### Main results, sensitivity analysis, and ranking from SUCRA

**Figure 5:**
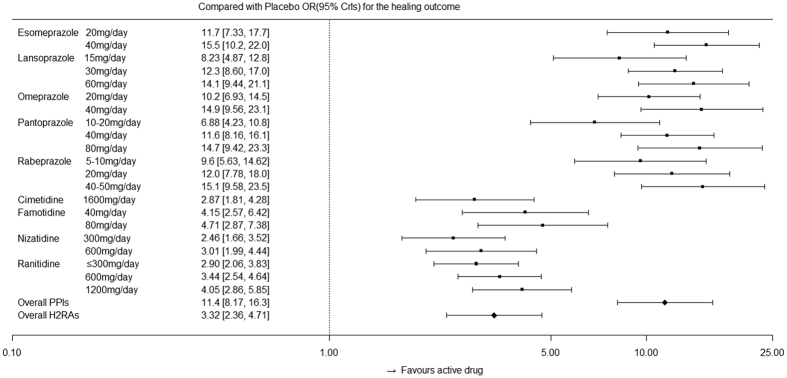
Effects of active drugs with different recommended doses and the overall effects from PPI and H_2_RA families compared with placebo for healing.

**Figure 6:**
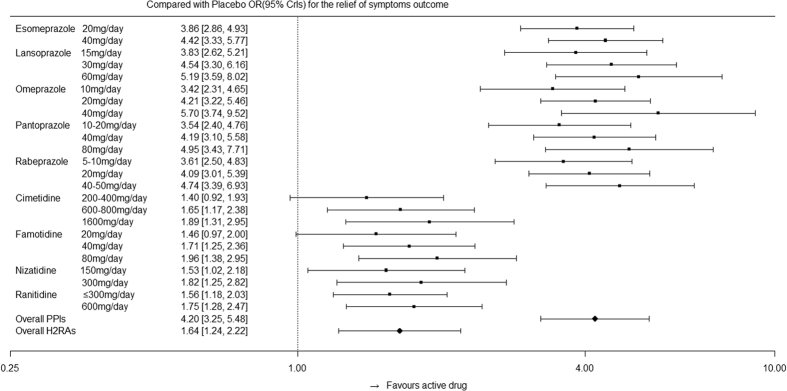
Effects of active drugs with different recommended doses and the overall effects from PPI and H_2_RA families compared with placebo for the relief of symptoms.

**Figure 7:**
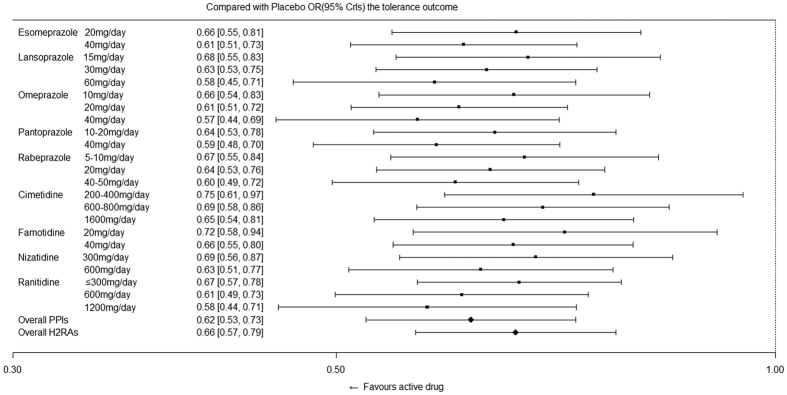
Effects of active drugs with different recommended doses and the overall effects from PPI and H_2_RA families compared with placebo for tolerance.

Figure 5 indicates the effects of active drugs with different recommended doses compared with those of placebo for the main outcomes, followed by the overall effect from the PPI and H_2_RA families. Figure 6 indicates the effects of active drugs with different recommended doses compared with those of placebo for relief of symptoms, whereas Fig. 7 indicates the effects of active drugs with different recommended doses compared with those of placebo for tolerance. Our results for all outcomes were separately placed in a trapezoid table that included the direct results and network results (Supplementary Tables S5, S6 and S7, respectively). Based on the overall results, the number of statistically significant effectiveness results was noticeably more than the tolerance outcome. For the main outcome, we identified features that indicated the curative effects of PPIs were strongly superior to the effects of H_2_RAs, and the high dose was superior to the low dose for the same intervention. There is a gap between the direct results and the network results at certain nodes; however, the majority of the 95% CrIs from the direct comparison results were covered by our network results, which demonstrated more conservation. In terms of symptom relief, the efficacy of the active drugs was significantly better than that of the placebo, as determined by the comparison between the PPIs and H_2_RAs (Fig. 6). Furthermore, inconsistency (0.107) accounted for 20.7% of the overall heterogeneity (0.517). In terms of tolerance, the comparison results among active drugs did not reach statistical significance; however, the tolerance was better than the placebo (Fig. 7).

Post hoc comparison results from the sensitivity analysis are presented (Supplementary Table S8). All results from all outcomes whose 95% CrIs substantially overlapped across base cases and sensitivity analyses were strongly stable according to our pre-specified design analyses. The one inconsistency node did not cause substantive changes to the results, and it further confirmed the reliability of our results. All ranks did not significantly change.

**Figure 8:**
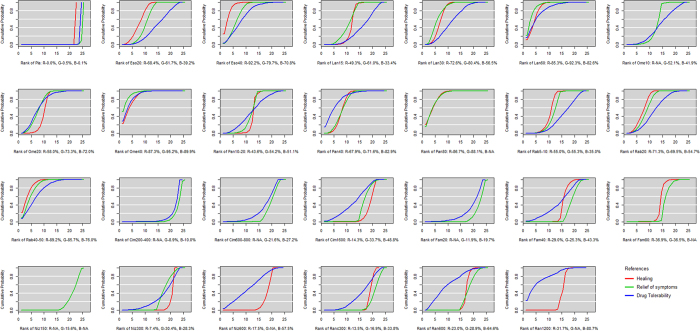
Ranking of all treatment drugs for healing, relief of symptoms, and tolerance outcomes. (All drug treatments were ranked according to their surface under the cumulative ranking (SUCRA) values. In ranking order for healing rates, from best to worst, the higher SUCRA scores demonstrate better effects. The red line indicates healing. In a similar manner, the green line indicates the relief of symptoms, and the blue line indicates tolerance. Eso20: esomeprazole at 20 mg per day, Eso40: esomeprazole at 40 mg per day, Lan15: lansoprazole at 15 mg per day, Lan30: lansoprazole at 30 mg per day, Lan60: lansoprazole at 60 mg per day, Ome10: omeprazole at 10 mg per day, Ome20: omeprazole at 20 mg per day, Ome40: omeprazole at 40 mg per day, Pan10–20: pantoprazole at 10–20 mg per day, Pan40: pantoprazole at 40 mg per day, Pan80: pantoprazole at 80 mg per day, Rab5–10: rabeprazole at 5–10 mg per day, Rab20: rabeprazole at 20 mg per day, Rab40–50: rabeprazole at 40–50 mg per day, Cim200–400: cimetidine at 200–400 mg per day, Cim600–800: cimetidine at 600–800 mg per day, Cim1600: cimetidine at 1600 mg per day, Fam20: famotidine at 20 mg per day, Fam40: famotidine at 40 mg per day, Fam80: famotidine at 80 mg per day, Niz150: nizatidine at 150 mg per day, Niz300: nizatidine at 300 mg per day, Niz600: nizatidine at 600 mg per day, Ran ≤300: ranitidine at ≤300 mg per day, Ran600: ranitidine at 600 mg per day, Ran1200: ranitidine at 1200 mg per day).

Figure 8 presents the ranking of all active drugs and placebo for all outcomes based on the SUCRA. Viewed as a whole, the PPI family ranked in front of the H_2_RA family on the effectiveness evaluation outcome. Most high doses were better than the low doses in the internal “nest” derived from different doses of single interventions for all outcomes. In the main outcome, esomeprazole at 40 mg per day (92.2%) from the PPI family ranked first, followed by rabeprazole at 40–50 mg per day (89.2%), Omeprazole at 40 mg per day (87.3%), pantoprazole at 80 mg per day (86.7%), and famotidine at 80 mg per day (36.9%) from the H_2_RA family. In the relief of symptom outcome, omeprazole at 40 mg per day (95.2%) from the PPI family ranked first, followed by lansoprazole at 60 mg per day (92.3%), pantoprazole at 80 mg per day (88.1%), and famotidine at 80 mg per day (36.5%) from the H_2_RA family. For drug tolerance, omeprazole at 40 mg per day (89.9%) from the PPI family ranked first, followed by pantoprazole at 40 mg per day (82.9%), lansoprazole at 60 mg per day (82.6%), and ranitidine at 1200 mg per day (80.7%) from the H_2_RA family. The placebo was the worst for all outcomes. All rankings exhibited strong stability in the sensitivity analyses.

### Quality of treatment effect estimates based on the GRADE system

For the main outcome, the quality of estimates substantially varied across comparisons within the network. All quality ratings of pair-wise comparisons from the network results, which were derived from quality ratings of direct comparisons and included 9 high, 16 moderate, 18 low, and 13 very low, had 10 high, 40 moderate, 91 low, and 91 very low (Supplementary Table S9). Moreover, 14 quality ratings of the network results had changed. The quality ratings from direct and indirect comparisons were low because of imprecision; however, the precision from network results was perfected, and the higher level was awarded to the quality of the network result, such as the comparison of pantoprazole 80 mg per day and pantoprazole 40 mg per day. The quality rating of direct evidence was moderate and indirect evidence was low for the comparison of lansoprazole at 15 mg per day and placebo; however, the inconsistency was identified via the “node-splitting” method. Thus, the quality rating of the network meta-analysis was clearly defined as low. The quality rating of esomeprazole at 40 mg per day against placebo was very low because of the disadvantage from indirect evidence and the lack of direct evidence; however, the quality ratings of pantoprazole 10–20 mg per day, pantoprazole 40 mg per day and ranitidine 1200 mg per day remained very optimistic.

## Discussion

In our network meta-analysis, we performed a series of adjusted hierarchical multi-regressions to investigate the effectiveness and tolerability of PPIs and H_2_RAs in the treatment of GERD for adults, thereby overcoming the major limitation of conventional pairwise meta-analysis^23^,^24^, with the ultimate aim to obtain current, reliable and high-quality evidence to influence the latest guideline development^8^. Moreover, we have provided rankings of all included drug interventions with various recommended doses from the guidelines, which may serve as a decision-making tool for clinicians^25^.

To better grasp the true nature of the drugs, we discussed relevant baseline and potential confounding factors that may influence the size of the treatment effect. First, our study indicated that most factors did not influence the size of the treatment effect in our main outcome, whereas the results for relevant coefficient factors indicated that there was a specific relationship between baseline and treatment effects. For example, a higher percentage of females was associated with a worse healing efficacy of endoscopy^26^. A concerning result regarding the confounding factor of pharmaceutical industry sponsorship unequivocally indicated that industry sponsorship biases study outcomes in favor of the sponsoring company’s product and may have resulted in an overestimation of the healing efficacy^27^. For the relief of symptom outcome, the inconsistency will be further discussed later. In the tolerance comparison, esomeprazole did not demonstrate a strong advantage among active interventions. Year of publication exhibited weak statistical significance (B = 0.028 (0.008, 0.049), P = 0.007); thus, all-cause discontinuation increases over time. Year is a comprehensive indicator, including the development of new drugs, improved efficacy and safety of drugs, as well as high-quality study design^28^. The most important reasons for discontinuation may be attributable to newly developed drugs with enhanced efficacy and improvements in endoscopy or a physician’s professional evaluation. Thus, the course of treatment may be reduced following professional evaluation by a clinical physician.

Bias refers to systematic error, which suggests that multiple replications of the same trial would not reach the same conclusion^29^,^30^. In general, randomized research designs are not subject to many biases that affect the weaker forms of evidence obtained from non-randomized studies; however, important deficits in the design, conduct, analysis, and reporting may lead to bias in randomized controlled trials^29^. According to the results from the pre-set selective design bias^31^, we determined that the results obtained from adjusting four selective design bias models were similar to the results obtained from the non-adjusted model for all outcomes. In the relief of symptom category, the PPI favored bias was significant, and the variance of the coefficient from the fixed coefficients model (1.681) was slightly higher than the exchangeable coefficient (1.589). Despite its lower global heterogeneity results, PPI favored bias exhibited specific advantages compared with the other design biases, as well as the non-adjusted bias model, and the models failed to adequately explain the sources of the inconsistencies. When the supplementary merger favored models were simultaneously used, the results demonstrated a substantial change. This finding suggested that an interaction effect between PPI favored bias and sponsored favored bias may exist^32^ because some studies exhibited antagonistic sponsored favored biases that weakened the efficacy of the PPI favored bias^33^. Compared with the other outcomes, the main and tolerance outcomes demonstrated that all design bias models had sufficient advantages compared with the non-adjusted model.

Consistency has been described as the relationship between direct and indirect sources of evidence for a single comparison in a network meta-analysis^34^. When inconsistency is identified in a statistical analysis, it may indicate a lack of transitivity. Few inconsistency nodes were identified for healing and tolerance based on the “node-splitting” method; however, our results based on the sensitivity analysis indicated that the removal of inconsistency nodes conferred no changes and demonstrated no clear advantage. The results for the relief of symptom outcome were negative; however, inconsistency comprised several nodes. Based on the statistical analysis, the results were obtained after using the “node-splitting” method to separate out pharmaceutical industry sponsorship. However, the results obtained from the adjustment of confounding factors and design bias suggest that pharmaceutical industry sponsorship did not affect the results in terms of the scale of statistical significance. Furthermore, the inconsistency excluded the awful drug with inconsistent nodes (lansoprazole at 15 mg per day). This finding suggested that the nature of the inconsistency was consistent with the boundary, which was falsely associated with pharmaceutical industry sponsorship on the surface, and inconsistency as part of the heterogeneity was widespread and difficult to identify among all comparisons^35^. The two main reasons for exaggerated clinical heterogeneity were that the relief of symptoms was evaluated using the standard of excessive subjective judgments from specialist physicians or patients and too many types of symptoms were mixed chaotic, such as heartburn, regurgitation, and other related symptoms^36^,^37^. We simultaneously considered that the number of esomeprazole was one important factor that led to the result deviation from clinical significance. Based on the cost of the guideline of NICE 2014^8^, it was determined that the cost of esomeprazole was more expensive than omeprazole. Considering these factors, the result of the relief of symptoms may require caution by clinicians. However, the ranking results following the addition of the inconsistency factors were not affected based on the hierarchical modeling approach that incorporated dose-related and PPI favored constraints.

Network meta-analyses may be used to analyze studies with multiple intervention groups, synthesize studies that make different comparisons of interventions and provide rankings for all interventions^25^. Rankings based on a network meta-analysis may provide important evidence for making clinical decisions^38^. In our main outcome, we provided more precise ranking results: esomeprazole at 40 mg per day from the PPI family ranked first, followed by rabeprazole at 40–50 mg per day and pantoprazole at 80 mg per day. Based on the GRADE system, the quality rating of esomeprazole at 40 mg per day against placebo was low, which was mainly a result of the lack of direct evidence and the disadvantage from the rationale of indirect evidence^39^ that the lower confidence rating of the two direct comparisons constitutes the confidence rating of the indirect comparison. Considering the clinical significance and SUCRA results, the full/standard doses (40 mg per day) of esomeprazole should be recommended as first-line treatments for GERD in adults based on 4–8 weeks of short-term therapy for healing. In a network meta-analysis of internal clinical guidelines (updated 2014)^8^, rabeprazole at 50 mg per day (extended-release, ER) was ranked first, followed by esomeprazole at 40 mg per day. However, the guidelines indicated that this result was based on one small trial; thus, it was subject to substantial uncertainty. Considering that more samples and more reliable methodologies were used in our study, we maintained that our results were more reliable in terms of healing for GERD in adults based on 4–8 weeks of short-term therapy. Our study simultaneously supplemented the lack of ranking evidence for guidelines, including that omeprazole at 40 mg per day had outstanding advantages for symptom relief and was well tolerated.

### Strengths and limitations

Direct-comparison methodology remains the most widely used research technique. However, when multiple interventions are investigated in one clinical question, a network meta-analysis may provide answers^38^. In this study, we used a Bayesian approach^40^ and pooled direct and indirect comparison data from 98 randomized studies that investigated the effects of numerous pharmacological interventions, specifically PPIs and H_2_RAs, for the treatment of GERD; moreover, we obtained a hierarchical ranking of their efficacy and tolerability profiles. Furthermore, the corresponding covariates and biases were adjusted and analyzed via the multiple meta-analysis method. From a clinical perspective, our analysis reinforces the existing evidence and both updates and supplements the evidence limited by potential confounding factors, such as the level of disease from endoscopy and symptoms. Considering the clinical significance, a hierarchical modeling approach that incorporated dose-related and PPI favored constraints was used to fully determine the potential advantages of both the PPI family and high-dose interventions. The GRADE system was applied in our main outcome to evaluate the quality of treatment effect and provide advice for clinicians. Thus, our findings are more applicable in the formulation of clinical practice guidelines.

Our study has several limitations. First, our meta-regression analyses used a single covariate to obtain adjusting results; future efforts that introduce multiple covariates to establish a high-quality and accurate model for further analyses are warranted^41^. Second, missing data present a threat to the validity of a randomized trial because the individuals with observed outcomes may not be representative of all individuals in the trial, and our study lacked corresponding evidence to demonstrate its impact on the overall results^42^. Third, additional samples and high quality randomized controlled trials are critical components in the production of high quality sources of evidence^29^. We look forward to additional research and superior methodology to update and perfect the current body of evidence.

## Conclusion

This study indicates that the full/standard doses (40 mg per day) of esomeprazole should be recommended as first-line treatments for GERD in adults based on 4–8 weeks of short-term therapy for healing. Moreover, the full/standard doses (40 mg per day) of omeprazole were associated with the relief of symptoms and were well tolerated. These findings may be applicable for the development of the latest clinical practice guidelines and should serve as a useful decision-making reference for clinicians.

## Methods

### Data Sources and Literature Searches

We searched PubMed, EMBASE, the Cochrane Library and ClinicalTrials.gov through September 1, 2016 for eligible randomized clinical trials that investigated PPIs or H_2_RAs for the treatment of GERD using Medical Subject Headings (MeSH) and text words. The search terms used for PubMed are presented in Supplementary Appendix S1. We also screened the reference lists of relevant published systematic reviews for additional studies and searched the US Food and Drug Administration (FDA) website for information regarding approvals.

### Study Selection

We included randomized, parallel-group clinical trials that investigated the efficacy and tolerability of PPIs, H_2_RAs and placebos. The participants included adult (≥18 years) patients with GERD. Our primary outcome was healing, measured via endoscopy. The secondary outcomes included the relief of symptoms and tolerance. Relief of symptoms (evaluation standard by specialist physicians or patients) was defined as the control and/or relief of GERD symptoms: overall assessments from symptoms, heartburn (defined as a burning discomfort or pain behind the breastbone), regurgitation (defined as a sensation of stomach contents rising into the throat or mouth), or other related symptoms. In cases of multiple report results for the relief of symptoms, the priority order was determined in advance: overall assessments from symptoms, heartburn, regurgitation and “other symptoms”. Tolerance was defined as any reason that caused discontinuation during the entire treatment duration. Studies that reported at least one of the three outcomes were eligible for inclusion. Based on the principles gained from the similarity of overall research^36^ and clinical applicability^9^, we designated the treatment periods for all outcomes as short-term therapy (4–8 weeks). If there were multiple nodes of data within 4–8 weeks, we selected the final time node as the outcome data. If the data were not in this range, the nodes could be no less than 1 week and no more than 12 weeks, and data from the closest setting time were extracted. The language of all included studies was limited to English.

The exclusion criteria were as follows: cross-over studies, GERD symptoms caused by chronic cough, asthma or simple laryngitis, Zollinger-Ellison syndrome, a primary motility disorder, esophageal stricture, Barrett’s esophagus, evidence of upper gastrointestinal malignancy, laryngopharyngeal reflux disease, and other severe concomitant disease. Duplicate published research and sample study arms of less than 30 were excluded. Based on the potential differences in molecular structures and drug efficacies of dexlansoprazole and lansoprazole, our study does not include dexlansoprazole^26^.

### Data Extraction and Disposal

Four authors (GLY, HW, JZY, and YPW) independently extracted relevant information regarding the study, including patient characteristics, interventions, comparisons, and outcomes. For missing data, particularly study design or outcomes, we contacted the original study authors for clarification. Intention-to-treat (ITT) data were used for all outcomes when possible; otherwise, per-protocol (PP) data were used^43^. To discuss the sources of heterogeneity/inconsistency and their influence on the results as a result of baseline and confounding factors, the area, gender (the percentage of females), age, level of disease from endoscopy and symptoms, degree of symptom relief, methodological quality score, year of publication, pharmaceutical industry sponsorship, ITT/PP and withdrawal rate were accounted for in the meta-regression analysis. The percentages of levels C and D from the Los Angeles grade (LA) (or the Savary-Miller criteria (SM: 3–4) or the Hetzel-Dent scale (HD: 3–4)) standard were defined to describe the covariation of the level of severe erosive esophagitis obtained via endoscopy. We gathered all doses per day as a unit, according to the recommended standards based on guidelines^8^.

Two authors (RXY and JZY) independently assessed the methodological quality of the included trials using the Cochrane Collaboration’s tool for risk of bias assessment^29^. We assessed the following domains: (i) random sequence generation; (ii) allocation concealment; (iii) blinding of participants and personnel; (iv) blinding of outcome assessors; (v) incomplete outcome data; (vi) selective reporting; and (vii) other sources of bias. Disagreements were resolved by discussion between the two authors or consultation with a third author (Joey S.W. K.) if necessary. For each methodological attribute included, we assigned the studies a rating of high, uncertain, or low quality. For each trial, we assigned one point for each “high quality” (or low risk) item to calculate the overall methodological quality score, which ranged from 0 (worst methodological quality) to 6 (best methodological quality). Using this information, we evaluated the distribution of the methodological qualities of different comparisons across the evidence network.

We subsequently extracted information regarding the funding sources of the included trials. Two potential funding sources were industry and non-industry (such as government, academic institutions, and other not-for-profit research organizations). The industry sources included private, for-profit pharmaceutical companies involved in research and development, manufacturing, or marketing sources. Most of the included trial publications contained a statement that delineated the funding sources; in cases in which the sponsorship information was not clearly documented, we checked the author affiliations and categorized the studies with industry affiliated authors as industry sponsored.

To investigate the presence of related potential bias^44^, we compared four categories of selective design bias for the existence of small-study effects: new favored bias, sponsored favored bias, high-dose favored bias, and PPI favored bias^8^,^24^,^45^. New favored bias was defined by considering the date of licensing for each treatment or from the date of publication of the first trial that evaluated a treatment for the investigated condition. Sponsored favored bias was defined by considering the profit relationship between drugs and industry sponsors. High-dose bias was defined by only considering a potentially favored high-dose intervention relation to a low-dose intervention in different doses from identity intervention. Relative to other doses (or the full/standard or low-dose) of a drug, the high-dose (or the double-dose) drug from the guidelines were unconditionally involved. Based on the related clinical evidence^31^,^32^ that the PPI family was more effective as an anti-reflux agent than the H_2_RA family for patients with persistent symptoms who are treated empirically for GERD, we included another design bias, which was referred to as the PPI favored bias. Finally, we considered a supplementary merger favored bias, which is related to two types of probable design bias, to further investigate the cumulative effects and interrelations of design bias. Thus, we assigned a label to each arm of the study to ensure that the natural attributes of “direction” bias for testing and adjusting the selective design bias did not affect the results.

### Statistical Analysis

Network meta-analyses provide reliable evidence for direct and indirect multiple-intervention comparisons^46^. We used the Bayesian hierarchical randomized model for all network meta-analyses to improve the accuracy of the results. Analyses were conducted using WinBUGS (version 1.4.3, MRC Biostatistics Unit, Cambridge, UK). In our network meta-analysis, we used non-informative priors with vague normal (mean 0, variance 10,000) and uniform (0–3) prior distributions for parameters, such as the means and standard deviations^46^. Various levels of prior distribution were applied in the sensitivity analyses. First, 10,000 simulations were performed; we subsequently generated an additional 50,000 simulations with three sets of different initial values and sheared the first 10,000 simulations as the burn-in period in our model. We used the Brooks-Gelman-Rubin statistical method to assess the model convergence^47^. Based on 50,000 simulations with 50 thin, the point estimate adopted the median of the posterior distribution, and the corresponding 95% credible intervals used the 2.5^th^ and 97.5^th^ percentiles of the posterior distributions, which were interpreted in a similar fashion as conventional 95% confidence intervals. We conducted all pairwise meta-analyses in random models using R 3.1.1 software. For all comparisons, a common heterogeneity parameter was assumed. All outcomes were calculated relative to the median effect sizes as odds ratios (ORs) with their corresponding 95% credible intervals (CrIs)^48^.

Based on the underlying assumption of transitivity in the network, conflicts may exist between pairwise comparisons and the distribution of effect modifiers^49^. An inconsistency between direct and indirect evidence suggests that transitivity is not apparent between the results^50^. The “node-splitting” method, which separates evidence for a particular comparison (node) into “direct” and “indirect” and may be applied to networks in which trial-level data are available^51^, was used. The standard criterion is that when the p value is less than 0.05, a significant disagreement between the direct and indirect evidence from the nodes is noted. When this disagreement was identified in a single node or several nodes, we eliminated a single comparison node or awful drug to compare the effects on the overall results in the sensitivity analyses. If the disagreement was widespread among the nodes, potential interference factors^41^ as the main source of inconsistency were checked and investigated. When the inconsistency could not be explained, we used a full set of design-by-treatment interaction models with random inconsistency effects to isolate the inconsistency^52^.

To better detect and control bias, which leads to result deviations from the truth^53^,^54^,^55^, we pre-set four design selective biases and conducted a multiple-treatment meta-regression for the quantitative analysis and adjusted for potential small-study effects using three models: the fixed coefficient model, exchangeable coefficients model, and consistent coefficients model^44^,^54^,^56^. To compare the trade-offs between model fits, we measured the total posterior residual deviance (PD, posterior mean of the deviance under a given model minus the deviance for the saturated model) along with the deviance information criterion (DIC, sum of the posterior mean of the residual deviance and the effective number of parameters), which is a summary statistic used to compare models in a Bayesian framework^57^, as well as heterogeneity/inconsistency (*σ*)^58^. Smaller DIC values are preferred, and in complexity across models, low DIC values reflect a better compromise between the models; a difference value from the DIC of less than 3 suggests that there is little to choose between the two models, and the differences between them are not considered important. We also calculated design bias coefficients as medians with their corresponding 95% CrIs. The standard criterion is that when 95% CrIs including 0 are reported, insignificant disagreement exists. Furthermore, based on the previously described adjusted results and the existing clinical advantages from PPI and high-dose interventions, a hierarchical modeling approach that incorporated dose-related and PPI favored constraints was established to optimally fit the data^59^,^60^.

We performed several sensitivity analyses to determine the stability of the baseline results and rank based on all interventions, as well as to further investigate potential reasons for heterogeneity/inconsistency^29^. The pre-specified analyses were as follows: analysis excluding single-blinded and unblinded studies^61^, excluding study arms with zero events^62^, excluding the discontinued drug (cimetidine), and excluding studies from China, considering the concerns of overall research quality^63^. To summarize the probabilities, we used the surface under the cumulative ranking curve (SUCRA) to provide a summary statistic for the cumulative ranking^64^. By definition, SUCRA values reflect the effectiveness of an intervention; thus, larger SUCRA scores imply more effective interventions^64^. To completely understand the quality of the evidence body, the GRADE (The Grading of Recommendations Assessment, Development and Evaluation) systems were applied to each comparison for the main outcome^39^,^65^. Based on the recommendation evidence of reliability and workability, the optimal recommendation evidence is subsequently presented by combining the GRADE system result and the SUCRA result^39^,^66^. The latest PRISMA extension statement for the reporting of systematic reviews and network meta-analyses was used^67^.

## Additional Information

**How to cite this article:** Zhang, C. *et al*. Effectiveness and Tolerability of Different Recommended Doses of PPIs and H_2_RAs in GERD: Network Meta-Analysis and GRADE system. *Sci. Rep.* **7**, 41021; doi: 10.1038/srep41021 (2017).

**Publisher's note:** Springer Nature remains neutral with regard to jurisdictional claims in published maps and institutional affiliations.

## Supplementary Material

Supplementary Information

Supplementary Table S5

Supplementary Table S6

Supplementary Table S7
