# Supplementary material for: Effectiveness and Tolerability of Different Recommended Doses of PPIs and H2RAs in GERD: Network Meta-Analysis and GRADE system
Source: Sci Rep. 2017 Jan 19;7:41021. doi: 10.1038/srep41021 (PMC5244481; doi:10.1038/srep41021)
Supplement: Supplementary Information [file srep41021-s1.pdf]

Supplementary information

Effectiveness and Tolerability of Different Recommended Doses of PPIs and H_2RAs in GERD: Network Meta-Analysis and GRADE System

Chao Zhang[1], Joey S.W. Kwong[2], Rui-Xia Yuan[1, 3], Hao Chen[4], Chang Xu[5], Yi-Pin Wang[1], Gong-Li Yang[6], Jin-Zhu Yan[1], Le Peng[1], Xian-Tao Zeng[1], Hong Weng[1], Jie Luo[1], Yu-Ming Niu[1,6]

[1]Center for Evidence-Based Medicine and Clinical Research, Taihe Hospital, Hubei University of Medicine, Shiyan 442000, Hubei Province, China; [2]Chinese Cochrane Center, Chinese Evidence-Based Medicine Center, West China Hospital, Sichuan University, Chengdu 610041, Sichuan Province, China; [3]Department of Epidemiology and Biostatistics, School of Public Health, Wuhan University and Global Health Institute, Wuhan University, Wuhan 430000, Hubei Province, China; [4]Center for Evidence-Based Medicine, Nanjing University of Chinese Medicine, Nanjing 210000, Jiangsu Province, China; [5]Department of Urology, Zhongnan Hospital of Wuhan University, Wuhan University, Wuhan 430000, Hubei Province, China; [6]Department of Gastroenterology, Taihe Hospital, Hubei University of Medicine, Shiyan 442000, Hubei Province, China.

Supplementary Appendix S1

Appendix S1. Search strategies

Supplementary Appendix S1

Search strategies

We will search the electronic database included the PubMed, Embase, Scopus, and the Cochrane Library published up to 1 Sep 2016, then addition to search the ClinicalTrials.gov and FDA website as supplement. Follow, we will provide the search strategies of PubMed electronic database.

PubMed:

#1 Gastroesophageal Reflux[mh]
#2 Gastric Acid Reflux
#3 Esophageal Reflux
#4 GERD
#5 Gastro-oesophageal Reflux
#6 Acid reflux
#7 Reflux
#8 RE
#9 Nonerosive reflux oesophagitis
#10 NERD
#11 #1 OR #2 OR #3 OR #4 OR #5 OR #6 OR #7 OR #8 OR #9 OR #10
#12 Proton pump inhibitors
#13 Esomeprazole
#14 Nexium
#15 #13 OR #14
#16 Lansoprazole
#17 2-(((3-Methyl-4-(2,2,2-trifluoroethoxy)-2-pyridyl)methyl)sulfinyl)benzimidazole
#18 Bamalite
#19 Lansol
#20 Lanzor
#21 Prezal
#22 Monolitum
#23 Opiren
#24 Prevacid
#25 Ogastro
#26 Pro Ulco
#27 Promeco
#28 Takepron
#29 Ulpax
#30 Zoton
#31 AG 1749
#32 AG-1749
#33 AG1749
#34 Agopton
#35 Ogast

#36 #17 OR #18 OR #19 OR #20 OR #21 OR #22 OR #23 OR #24 OR #25 OR #26 OR #27 OR #28 OR #29 OR #30 OR #31 OR #32 OR #33 OR #34 OR #35
#37 Omeprazole
#38 Prilosec
#39 H 168-68
#40 H 168 68
#41 H 16868
#42 #37 OR #38 OR #39 OR #40 OR #41
#43 Pantoprazole
#44 SK and F 96022
#45 SKF-96022
#46 SK and F-96022
#47Protonix
#48 BY 1023
#49 BY-1023
#50 #43 OR #44 OR #45 OR #46 OR #47 OR #48 OR #49
#51 Rabeprazole
#52 2-((4-(3-methoxypropoxy)-3-methylpyridin-2-yl)methylsulfinyl)-1H-benzimidazole
#53 Dexrabeprazole
#54 E 3810
#55 E3810
#56 Pariet
#57 Aciphex
58 LY-307640
#59 LY 307640
#60 LY307640
#61 #51 OR #52 OR #53 OR #54 OR #55 OR #56 OR #57 OR #58 OR #59 OR #60
#62 Histamine H2 antagonists
#63 Receptor Blockaders, H2
#64 Blockaders, Histamine H2 Receptor
#65 Histamine H2 Receptor Blockaders
#66 Histamine H2 Blockers
#67 Blockers, Histamine H2
#68 H2 Blockers, Histamine
#69 Receptor Antagonists, Histamine H2
#70 Histamine H2 Receptor Antagonists
#71 Antagonists, Histamine H2
#72 H2 Antagonists, Histamine
#73 H2 Receptor Blockaders
#74 Blockaders, H2 Receptor
#75 Antihistaminics, H2
#76 H2 Antihistaminics
#77 H2RAs
#78 #62 OR #63 OR #64 OR #65 OR #66 OR #67 OR #68 OR #69 OR #70 OR #71 OR #72 OR

#73 OR #74 OR #75 OR #76 OR #77
#79 Cimetidine
#80 Eureceptor
#81 Altramet
#82 SK and F-92334
#83 SK and F 92334
#84 SK and F92334
#85 SKF-92334
#86 SKF 92334
#87 SKF92334
#88 Tagamet
#89 Biomet
#90 Histodil
#91 Biomet400
#92 N-Cyano-N'-methyl-N"-(2-(((5-methyl-1H-imidazol-4-yl)methyl)thio)ethyl) guanidine
#93 #79 OR #80 OR #81 OR #82 OR #83 OR #84 OR #85 OR #86 OR #87 OR #88 OR #89 OR #90 OR #91 OR #92
#94 Famotidine
#95 YM-11170
#96 YM 11170
#97 YM11170
#98 Pepcid
#99 Famotidine Hydrochloride
#100 MK-208
#101 MK 208
#102 MK208
#103 #94 OR #95 OR #96 OR #97 OR #98 OR #99 OR #100 OR #101 OR #102
#104 Nizatidine
#105
N-(2-(((2-((Dimethylamino)methyl)-4-thiazolyl)methyl)thio)ethyl)-N'-methyl-2-nitro-1,1-ethe#100 nediamine
#106 LY-139037
#107 LY 139037
#108 LY139037
#109 Axid
#110 #104 OR #105 OR #106 OR #107 OR #108 OR #109
#111 Ranitidine
#112
N(2-(((5-((Dimethylamino)methyl)-2-furanyl)methyl)thio)ethyl)-N'-methyl-2-nitro-1,1-ethenediamine
#113 Ranitidin
#114 Biotidin
#115 Ranisen
#116 Sostril

#117 Zantac
#118 Zantic
#119 AH-19065
#120 AH 19065
#121 AH19065
#121 #111 OR #112 OR #113 OR #114 OR #115 OR #116 OR #117 OR #118 OR #119 OR #120 OR #121
#122 #12 OR #15 OR #36 OR #42 OR #50 OR #61 OR #78 OR #93 OR #103 OR #110 OR #121
#123 randomized controlled trial [pt]
#124 controlled clinical trial [pt]
#125 randomized controlled trials [mh]
#126 random allocation [mh]
#127 double-blind method [mh]
#128 single-blind method [mh]
#129 clinical trial [pt]
#130 clinical trials [mh]
#131 "clinical trial" [tw]
#132 #32 OR #33 OR #34 OR #35 OR #36 OR #37 OR #38 OR #39 OR #40
#134 singl* [tw]
#135 doubl* [tw]
#136 trebl* [tw]
#137 tripl* [tw]
#138 #134 OR #135 OR #136 OR #137
#139 mask* [tw]
#140 blind* [tw]
#141 #139 OR #140
#142 #138 AND #141
#143 -placebos [mh]
#144 placebo* [tw]
#145 random* [tw]
#146 research design [mh:noexp]
#147 comparative study [pt]
#148 evaluation studies [mh]
#149 follow-up studies [mh]
#150 prospective studies [mh]
#151 control* [tw]
#152 prospective* [tw]
#153 volunteer* [tw]
#154 #143 OR #144 OR #145 OR #146 OR #147 OR #148 OR #149 OR #150 OR #151 OR #152 OR #153
#155 #132 OR #142 OR #154
#156 #11 AND #122 AND #155

Supplementary eTables Legends

Supplementary Table S1. Summary of included clinical trials and patient characteristics

Supplementary Table S2. Adjusting the confounding factors in all outcomes

Supplementary Table S3. Adjusting four selective design biases and model parameters for all outcomes

Supplementary Table S4. The results of compare models in all outcomes

Supplementary Table S5. The network results and the pairwise comparison results for healing **(Separate Excel documents)**

Supplementary Table S6. The network results and the pairwise comparison results for relief of symptoms **(Separate Excel documents)**

Supplementary Table S7. The network results and the pairwise comparison results for tolerance **(Separate Excel documents)**

Supplementary Table S8. The baseline results for the network analysis comprising sensitivity analyses

Supplementary Table S9. Estimates of effects and quality ratings for comparison of recommended doses of different drugs from the main outcome (healing) based on the GRADE system

Supplementary Table S1 Summary of included clinical trials and patient characteristics

Study	Year	County	Drugs, dose, usage	Total (female)	Age (Mean ± SD or range)	Level of disease from endoscopy or symptoms	Mean courses of disease	Follow-up from start of treatment (weeks)	Might be sponsor drugs	ITT/PP	Healing	Relief of symptoms	Tolerance
Kim[1]	2015	Korea	Omeprazole 20mg QD; Famotidine 20mg BID	167(46); 162(48)	50(20-74); 53(24-74)	Reflux esophagitis grade:A-D	≥ 2 weeks	8	Omeprazole	ITT	126/147; 92/145	87/147; 58/144	10/147; 10/147
Peura a[2]	2014	USA	Placebo; Esomeprazole 20mg QD	163(95); 168(104)	45.9±12.6; 43.6±12.2	Heartburn: Mild; Moderate; Severe	≥ 4 weeks	2	Esomeprazole	ITT	NA	54/163; 77/168	11/169; 8/171
Peura b[2]	2014	USA	Placebo; Esomeprazole 20mg QD	158(82); 162(86)	42.8±13.2; 41.6±14.0	Heartburn: Mild; Moderate; Severe	≥ 4 weeks	2	Esomeprazole	ITT	NA	52/158; 78/162	19/171; 19/170
Moraes-Filho[3]	2014	Brazil	Esomeprazole 40mg QD; Pantoprazole 40mg QD	288(144); 290(162)	42.3±12.6; 43.1±11.5	LA: A-D	NA	8	Esomeprazole; Pantoprazole	ITT	253/279; 246/284	227/264; 252/275	85/288; 71/290
Cho[4]	2012	Korea	Pantoprazole 20mg QD; Pantoprazole 40mg QD	67(22); 62(13)	51.2±12.6; 49.5±13.3	LA: A-C	NA	8	None	PP	63/67; 60/62	NA	8/75; 12/74
Kobeissy[5]	2012	USA	Ranitidine 150mg BID; Rabeprazole 10mg BID	39(23); 44(28)	45.1±15.3; 45.4±15.2	NA	3.9(4.2); 4.7(7.8) years	4	None	ITT	NA	17/39; 20/44	3/39; 4/44
Tan[6]	2011	Hong Kong	Placebo; Esomeprazole 40mg QD	175(123)	48.5; 48.3	A Chinese GerdQ reflux score≥12	NA	8	Esomeprazole	ITT	NA	29/78; 44/77	12/90; 8/85
Laine a[7]	2011	Mix	Rabeprazole 50mg QD; Esomeprazole 40mg QD	524(202); 531(206)	48.0±13.4; 49.0±13.1	LA: C,D	≥ 3 months	8	Rabeprazole	ITT	419/524; 398/531	253/524; 256/531	45/524; 40/531
Laine b[7]	2011	Mix	Rabeprazole 50mg QD; Esomeprazole 40mg QD	528(179); 537(195)	51.0±12.8; 49.1±13.4	LA: C,D	≥ 3 months	8	Rabeprazole	ITT	409/528; 421/537	281/528; 282/537	43/528; 42/537
Kinoshita[8]	2011	Japan	Placebo; Rabeprazole 5mg QD; Rabeprazole 10mg QD	91(51); 93(55); 101(51)	49.7±17.0; 46.3±15.3; 46.8±15.7	Heartburn: Mild; Moderate; Severe	NA	4	Rabeprazole	ITT	NA	31/91; 44/93; 56/101	5/93; 5/93; 4/102
Ohara[9]	2010	Japan	Placebo; Famotidine 20mg BID;	158(32); 150(38)	52.9; 52.6	LA: A,B	NA	8	None	PP	14/145; 86/140	NA	24/169; 23/163
Johnson[10]	2010	USA	Placebo; Esomeprazole 20mg QD	125(85); 137(89)	46.8±12.9; 47.0±11.7	NA	≥ 3 months	4	Esomeprazole	ITT	NA	9/125; 45/137	8/133; 6/143

Zheng[11]	2009	China	Omeprazole 20mg QD; Pantoprazole 40mg QD; Lansoprazole 30mg QD; Esomeprazole 40mg QD	68(35); 69(35); 69(34); 68(35)	57.9±14.1; 57.8±13.2; 58.1±13.0; 57.4±12.8	LA: A-D	NA	8	None	PP	57/65; 60/67; 61/67; 62/65	NA	3/68; 2/69; 2/69; 3/68
Peura[12]	2009	USA	Placebo; Lansoprazole 15mg QD; Lansoprazole 30mg QD	277(181); 291(173); 284(183)	47.4±13.7; 48.2±14.5; 48.8±14.1	Heartburn: Mild; Moderate; Severe	NA	2	Lansoprazole	ITT	NA	82/277; 145/291; 145/284 *	12/277; 13/291; 13/284
Kushner a[13]	2009	USA	Placebo; Lansoprazole 15mg QD	282(182); 282(178)	48.1(13.8); 46.3(13.8)	Heartburn: Mild; Moderate; Severe	4.0(1.6); 4.1(1.5) years	2	Lansoprazole	ITT	NA	NA	11/282; 13/282
Kushner b[13]	2009	USA	Placebo; Lansoprazole 15mg QD	282(190); 288(190)	47.6(13.2); 49.5(14.1)	Heartburn: Mild; Moderate; Severe	4.0(1.5); 4.0(1.5) years	2	Lansoprazole	ITT	NA	NA	13/282; 12/288
Eggleston[14]	2009	Australia	Rabeprazole 20mg QD; Esomeprazole 20mg QD; Esomeprazole 40mg QD	464(264); 459(208); 469(208)	45.8±0.68; 46.2±0.69; 48±0.68	NA	≥ 3 months	4	Rabeprazole; Esomeprazole	ITT	NA	405/464; 414/459; 418/469	69/464; 59/459; 63/469
Uemura[15]	2008	Japan	Placebo; Omeprazole 10mg QD; Omeprazole 20mg QD	92(19); 96(49); 93(40)	42.4±15.4; 44.4±16.2; 43.8±16.4	Heartburn: Mild; Moderate; Severe	NA	4	Omeprazole	ITT	NA	22/95; 44/96; 43/93	7/95; 3/96; 3/93
Hongo[16]	2008	Japan	Placebo; Famotidine 20mg QD; Famotidine 40mg QD	177(97); 177(97); 174(87)	47.3; 47.4; 46.3	Heartburn: Mild	NA	8	Famotidine	ITT	NA	106/177; 120/177; 132/174	11/177; 9/177; 9/174
Pilotto[17]	2007	Italy	Omeprazole 20mg QD; Pantoprazole 40mg QD; Lansoprazole 30mg QD; Rabeprazole 20mg QD	80(36); 80(41); 80(44); 80(43)	77.9±6.4; 76.8±6.1; 77.8±9.2; 77.0±9.5	SM: 1-4	NA	8	None	ITT	60/80; 68/80; 72/80; 71/80	64/80; 62/80; 77/80; 75/80	6/80; 3/80; 5/80; 5/80
Mönnikes[18]	2007	Germany	Pantoprazole 20mg QD; Esomeprazole 20mg QD	263(159); 266(167)	51.2±14.2; 52.6±15.4	LA: A-D	NA	4	Pantoprazole; Esomeprazole	ITT	NA	211/263; 211/266	41/263; 37/266
Glatzel[19]	2007	Germany	Pantoprazole 40mg QD; Esomeprazole 40mg QD	284(125); 277(136)	52.6±14.5; 54.0±14.3	LA: A-D	NA	4	Pantoprazole; Esomeprazole	ITT	NA	NA	40/284; 45/277
Bardhan[20]	2007	Germany	Pantoprazole 40mg QD; Esomeprazole 40mg QD	288(139); 293(139)	53±14; 54±14	LA: A-D	NA	8	Pantoprazole; Esomeprazole	ITT	248/288; 243/293	222/288; 220/293	NA
Bytzer[21]	2006	Europe	Omeprazole 20mg QD; Rabeprazole 20mg QD	359(162); 358(181)	51.7(19-81); 50.6(18-87)	LA: A-D	84.2(3.3-729.3); 73.7(3.1-626.4) months	1	Omeprazole; Rabeprazole	ITT	NA	NA	178/359; 170/358
Lightdale[22]	2006	USA	Esomeprazole 20mg QD; Omeprazole 20mg QD	588(216); 588(212)	44.7(13.2); 45.3(13.0)	LA: A-D	NA	8	Esomeprazole;	PP	508/588;	NA	9/588; 10/588

									Omeprazole		484/588		
Schmitt[23]	2006	USA	Esomeprazole 40mg QD; Omeprazole 20mg QD	576(230); 572(237)	47.1(13.3); 46.2(13.6)	LA: A-D	NA	8	Esomeprazole; Omeprazole	PP	501/576; 491/572	NA	18/576; 10/572
Pace[24]	2005	Italy	Omeprazole 20mg QD; Rabeprazole 20mg QD	272(88); 277(87)	47.1±14.9; 47.4±14.2	SM: 0-3	56.6(67.2); 51.5(59.0) months	8	None	PP	231/237; 228/237	NA	22/277; 25/283
Kahrilas[25]	2005	USA	Placebo; Rabeprazole 20mg QD	126(86); 126(89)	44.0±0.99; 43.1±1.04	Modified HD: 0,1	7.4(0.68); 7.8(0.63) years	4	None	ITT	NA	24/126; 66/126	15/132; 14/129
Johnson[26]	2005	USA	Placebo; Esomeprazole 20mg QD; Esomeprazole 40mg QD	221(131); 220(132); 209(125)	46.5±13.6; 46.8±14.0; 46.3±14.6	Heartburn: Mild; Moderate; Severe	≥ 3 months	4	Esomeprazole	ITT	NA	18/221; 102/220; 98/209	17/229; 8/226; 8/220
Fock[27]	2005	Asia	Rabeprazole 10mg QD; Esomeprazole 20mg QD	63(25); 64(37)	39.3±11.2; 38.4±10.0	NA	3.2(4.2); 3.9(4.7) years	4	None	ITT	NA	37/63; 27/64	23/63; 30/64
Fennerty[28]	2005	USA	Esomeprazole 40mg QD; Lansoprazole 30mg QD	498(171); 501(168)	47.3±13.2; 47.1±12.9	LA: C,D	NA	8	Esomeprazole	ITT	386/498; 367/501	344/478; 307/483	32/499; 30/502
Van Zyl[29]	2004	Germany and South Africa	Ranitidine 300mg QD; Pantoprazole 20mg QD	171(86); 167(81)	49.7±14.2; 48.1±15.0	NA	≥ 3 months	4	Pantoprazole	ITT	NA	114/167; 74/171	14/171; 18/167
Gillessen[30]	2004	Germany	Pantoprazole 40mg QD; Esomeprazole 40mg QD	113(49); 114(57)	53±15; 54±14	NA	NA	8	None	ITT	69/113; 70/114	57/113; 54/114	19/113; 11/114
Armstrong a[31]	2004	Canada, England and Ireland	Omeprazole 20mg QD; Esomeprazole 20mg QD; Esomeprazole 40mg QD	434(247); 423(240); 425(242)	48.3(19-78); 48.0(18-80); 48.4(19-79)	NA	≥ 6 months	4	Omeprazole; Esomeprazole	ITT	NA	252/434; 256/423; 241/425	NA
Armstrong b[31]	2004	France, Germany and	Omeprazole 20mg QD; Esomeprazole 40mg QD	346(190); 347(193)	50.0(19-83); 50.6(21-77)	NA	≥ 6 months	4	Omeprazole; Esomeprazole	ITT	NA	235/346; 244/347	NA

		Switzerland											
Armstrong c[31]	2004	Denmark, Finland, Norway and Sweden	Omeprazole 20mg QD; Esomeprazole 20mg QD	334(166); 336(161)	48.5(19-79); 48.5(20-79)	NA	≥ 6 months	4	Omeprazole; Esomeprazole	ITT	NA	199/334; 208/336	NA
Scholten[32]	2003	Germany	Pantoprazole 40mg QD; Esomeprazole 40mg QD	112(43); 105(48)	54.7±15.1; 52.6±13.8	LA: B,C	NA	5	Pantoprazole; Esomeprazole	ITT	NA	80/112; 74/105 *	13/112; 10/105
Katz a[33]	2003	USA	Placebo; Esomeprazole 20mg QD; Esomeprazole 40mg QD	124(62.9); 121(59.5); 123(61.0)	46(19-82); 47(21-75); 47(20-78)	Heartburn: Mild; Moderate; Severe	≥ 6 months	4	None	ITT	NA	17/123; 41/121; 41/124 *	6/123; 9/121; 9/124
Katz b[33]	2003	USA	Placebo; Esomeprazole 20mg QD; Esomeprazole 40mg QD	118(57.6); 113(69.9); 118(64.4)	47(21-77); 46(19-75); 45(19-77)	Heartburn: Mild; Moderate; Severe	≥ 6 months	4	None	ITT	NA	14/118; 47/113; 43/118 *	12/118; 11/113; 4/118
Kŏrner[34]	2003	Europe	Omeprazole 40mg QD; Pantoprazole 40mg QD	332(146); 337(121)	53.8±14.6; 53.7±14.3	SM: 2,3	NA	8	Omeprazole, Pantoprazole	ITT	220/332; 220/337	250/270; 257/282	4/332; 3/337
Kao[35]	2003	Taiwan	Omeprazole 40mg QD; Esomeprazole 20mg QD	50(15); 50(16)	49.2; 49.8	LA: A,B	NA	4	None	ITT	NA	34/50; 23/50	4/50; 5/50
Adachi[36]	2003	Japan	Omeprazole 20mg QD; Rabeprazole 20mg QD	30(15); 30(15)	67.3±11.7; 65.3±13.0	LA: A-D	NA	8	None	ITT	NA	NA	2/30; 2/30
Mulder[37]	2002	Netherland	Omeprazole 20mg QD; Pantoprazole 40mg QD; Lansoprazole 30mg QD	151(63); 154(60); 156(66)	51.6±15.0; 51.2±14.4; 50.8±14.5	Modified SM: 1-4	NA	8	None	ITT	NA	131/151; 125/154; 139/156 *	7/151; 5/154; 11/156
Miner[38]	2002	USA	Placebo; Rabeprazole 10mg QD; Rabeprazole 20mg QD	70(46); 65(37); 68(43)	46.1±1.2; 44.4±1.5; 45.5±1.3	HD: 0,1	NA	4	Rabeprazole	ITT	NA	22/68; 36/64; 38/67	NA
Meneghelli[39]	2002	Brazil	Ranitidine 150mg BID; Pantoprazole 40mg QD	128(40); 128(48)	47.0(21-74); 46.5(19-82)	SM: 2,3	NA	8	Ranitidine, Pantoprazole	ITT	52/128; 96/128	60/113; 99/109 *	18/128; 21/128
Kovacs[40]	2002	USA	Nizatidine 150mg BID; Pantoprazole 20mg BID; Pantoprazole 40mg BID	221(66)	50.1±13.4; 47.8±12.9; 49.4±13.8	HD 2-4	NA	8	Nizatidine, Pantoprazole	ITT	29/70; 57/72; 58/70	NA	NA
Holtmann[41]	2002	Europe	Omeprazole 40mg QD; Rabeprazole 20mg QD	116(36); 118(44)	51.0(18-79);	SM: 2,3	2.51(0-19);	8	Omeprazole,	ITT	NA	NA	9/126; 12/125

					52.9(20-80)		2.55(0-21) years		Rabeprazole				
Castell[42]	2002	USA	Lansoprazole 30mg QD; Esomeprazole 40mg QD	2627(1116); 2624(1120)	47.4±13.1; 47.0±13.0	LA: A-D	NA	4	None	ITT	2324/2617; 2430/2624	1575/2627; 1650/2624 *	NA
Richter[43]	2001	USA	Omeprazole 20mg QD; Lansoprazole 30 mg QD	1756(772); 1754 （747)	46.9±13.6; 47.8±13.8	SM: 1-4	NA	8	Omeprazole, Lansoprazole	ITT	NA	1457/1756; 1479/1754	78/1756; 75/1754
Richter[44]	2001	USA	Omeprazole 20mg QD; Esomeprazole 40mg QD	1209(449); 1216(496)	NA	LA: A-D	NA	8	Omeprazole, Esomeprazole	ITT	1018/1209; 1139/1216	929/1209; 1036/1216	54/1209; 55/1216
Paul[45]	2001	USA	Placebo; Nizatidine 75mg BID	496(262); 498(277)	43.5(16-81); 42.3(16-81)	NA	≥ 3 months	2	None	ITT	NA	119/496; 184/498 *	NA
Kaspari[46]	2001	Germany	Ranitidine 150mg BID; Pantoprazole 20mg QD	175(88); 181(97)	48.8±13.2; 48.5±13.8	SM: 0,1	≥ 3 months	4	Ranitidine, Pantoprazole	ITT	NA	91/132; 124/141	42/175; 37/181
Bardhan[47]	2001	Europe	Omeprazole 20mg QD; Pantoprazole 20mg QD	161(58); 166(79)	45.2±14.4; 44.6±13.3	SM: 1	NA	8	None	ITT	142/161; 134/166	140/161; 138/166	30/161; 33/166
Armstrong[48]	2001	Canada	Nizatidine 150mg BID; Pantoprazole 40 mg QD	102(51); 106(49)	47.6±14.1; 47.1±14.0	SM: 0-3	≥ 6 months	4	Nizatidine	ITT	73/109; 95/111	37/109; 67/111 *	15/109; 10/111
Van Zyl[49]	2000	South Africa	Ranitidine 300mg QD; Pantoprazole 20mg QD	100(44); 101(40)	18-82	NA	NA	8	Ranitidine, Pantoprazole	ITT	69/100; 84/101	45/89; 74/88	16/100; 13/101
Richter[50]	2000	USA	Placebo; Omeprazole 10mg QD; Omeprazole 20mg QD	123(56); 118(53); 118(57)	49.7; 50.0; 49.5	NA	≥ 12 months	4	Omeprazole	ITT	NA	28/123; 58/118; 87/118 *	NA
Richter[51]	2000	USA	Placebo; Pantoprazole 10mg QD; Pantoprazole 20mg QD; Pantoprazole 40mg QD	82(29); 174(63); 174(59); 173(52)	48.3±14.0; 49.6±13.9; 48.7±12.4; 49.3±13.6	HD: 1-4	NA	8	Omeprazole, Pantoprazole	ITT	27/82; 102/174; 135/174; 152/173	NA	NA
Kahrilas[52]	2000	USA	Omeprazole 20mg QD; Esomeprazole 20mg QD; Esomeprazole 40mg QD	650(251); 656(265); 654(270)	46.5±13.5; 45.3±13.3; 44.8±13.0	LA: A-D	NA	8	Omeprazole, Esomeprazole	ITT	565/650; 590/656;	357/624; 382/626; 402/621	51/650; 60/654; 48/656

											615/654		
Fass[53]	2000	USA	Omeprazole 40 mg QD; Lansoprazole 30mg BID	46(2); 44(4)	57.8±14.7; 57.8±12.1	NA	≥ 3 months	6	Omeprazole, Lansoprazole	ITT	NA	8/46; 10/44 *	4/50; 2/46
Farley[54]	2000	UK	Ranitidine 150mg QID; Rabeprazole 20mg QD	169(56); 169(51)	50.4±14.2; 51.4±14.9	Modified HD: 2-4	NA	8	Ranitidine, Rabeprazole	ITT	112/169; 146/167	108/158; 127/161	12/169; 15/167
Delchier[55]	2000	Europe	Omeprazole 20mg QD; Rabeprazole 10mg QD; Rabeprazole 20mg BID	103(63); 103(72); 104(57)	53±15.1; 52±14.3; 55±15.7	HD: 2-4	NA	8	Omeprazole, Rabeprazole	ITT	97/103; 94/103; 95/104	NA	17/104; 21/103; 25/104
Richter[56]	1999	USA	Placebo; Lansoprazole 15mg QD; Lansoprazole 30mg QD	43(25); 80(41); 86(47)	46.4±12.5; 44.9±14.9; 43.4±13.5	NA	NA	4-8	Lansoprazole	ITT	NA	NA	7/43; 5/80; 8/86
Pappa[57]	1999	USA	Placebo；Ranitidine 25mg QID; Ranitidine 75mg QID	494(177); 504(178); 491(156)	47.3(18-83); 46.7(18-84); 47.4(18-87)	NA	≥ 3 months	2	Ranitidine	ITT	NA	197/470; 245/480; 275/482	24/494; 24/504; 9/491
Pappa[58]	1999	USA	Placebo; Ranitidine 25mg QD; Ranitidine 75mg QD; Ranitidine 125mg QD	143(48); 144(49); 143(47); 147(58)	41.0±1.06; 42.0±1.09; 39.5±1.09; 40.8±1.02	NA	≥ 3 months	4	Ranitidine	ITT	NA	72/143; 97/144; 102/143; 104/147	3/143; 3/144; 4/143; 5/147
Maton[59]	1999	USA	Ranitidine 150mg BID; Omeprazole 20mg QD	161(90); 156(91)	45.8; 44.7	NA	≥ 6 months	8	Ranitidine, Omeprazole	ITT	NA	71/144; 104/148	15/161; 8/156
Jones[60]	1999	UK	Omeprazole 10mg QD; Lansoprazole 15mg QD	279(143); 283(143)	46; 47	NA	NA	4	Omeprazole, Lansoprazole	ITT	NA	142/279; 167/283 *	50/279; 48/283
Jansen[61]	1999	Netherland	Ranitidine 300mg BID; Lansoprazole 30mg QD	65(24); 68(26)	53.3±13.7; 53.7±14.8	NA	NA	8	None	ITT	43/65; 62/68	43/65; 60/68	2/65; 7/68
Festen[62]	1999	Netherland	Ranitidine 300mg BID; Omeprazole 20mg QD	224(109); 222(104)	49.8±13.9; 51.1±14.5	SM: 1,2	NA	8	Ranitidine, Omeprazole	ITT	NA	112/224; 165/222	29/224; 28/222
Galmiche[63]	1998	France and Germany	Placebo; Cimetidine 200mg QD; Ranitidine 75mg QD	270(161); 515(306); 504(285)	49.9±0.9; 50.6±0.7; 48.0±0.6	SM: 0,1	5.5±0.4; 5.6±0.3; 5.1±0.3 years	2	Cimetidine, Ranitidine	ITT	NA	208/270; 417/515; 423/504	52/270; 83/515; 64/504
Earnest[64]	1998	USA	Placebo; Lansoprazole 15mg QD; Lansoprazole	NA	NA	NA	NA	8	Lansoprazole	ITT	31/66; 60/72;	NA	7/66; 6/72; 6/71;

			30mg QD; Lansoprazole 60mg QD								62/71; 70/79		8/79
Dettmer[65]	1998	Germany	Ranitidine 300mg QD; Pantoprazole 20mg QD	104(39); 105(41)	54(21-82); 47(19-80)	SM: 1	NA	8	Pantoprazole	ITT	63/104; 78/105	55/84; 68/85	18/104; 18/105
Venables[66]	1997	UK	Ranitidine 150mg BID; Omeprazole 10mg QD; Omeprazole 20mg QD	326(50); 338(51); 320(48)	50±14; 51±15; 51±14	Modified SM: 0-1	NA	4	Ranitidine, Omeprazole	ITT	NA	131/326; 167/338; 200/330	NA
Galmiche[67]	1997	Europe	Omeprazole 10mg QD; Omeprazole 20mg QD	144(89); 141(78)	51±15; 50±16	Heartburn: Mild; Moderate; Severe	≥ 3 months	8	Omeprazole	ITT	NA	102/144; 112/141	NA
Bate[68]	1997	UK	Cimetidine 400mg QD; Omeprazole 20mg QD	109(37); 112(48)	46±14; 49±13	SM: 0-3	NA	4	Cimetidine, Omeprazole	ITT	NA	34/109; 74/112	22/109; 14/112
Silver[69]	1996	USA	Placebo; Ranitidine 300 mg BID; Ranitidine 150mg QID	251(81); 260(83); 261(91)	47.9; 48.6; 48.8	HD: 2-4	NA	8	Ranitidine	ITT	79/217; 118/230; 145/235	NA	51/251; 38/260; 40/261
Rensburg[70]	1996	Germany	Pantoprazole 40mg QD; Pantoprazole 80mg QD	97(40); 95(38)	46(18-88); 46(21-89)	SM: 2,3	NA	8	Pantoprazole	ITT	82/97; 82/95	83/86; 85/87	NA
Mee[71]	1996	UK, Ireland	Lansoprazole 30mg QD; Omeprazole 20mg QD	NA	18-80	SM: 1-4	NA	8	None	ITT	226/300; 216/304	NA	75/300; 75/304
Mccarty-Dawson[72]	1996	USA	Cimetidine 800mg BID; Ranitidine 150mg BID; Ranitidine 150mg QID	231(89); 236(99); 229(84)	46.8; 48.4; 46.5	HD: 2-4	NA	8	Cimetidine, Ranitidine	ITT	120/231; 132/236; 153/229	NA	43/231; 32/236; 20/229
Castell[73]	1996	UK	Placebo; Omeprazole 20mg QD; Lansoprazole 15mg QD; Lansoprazole 30mg QD	213(71); 431(171); 218(73); 421(133)	47.6(18-80); 47.5(19-78); 45.8(20-82); 48.6(18-84)	HD: 2-4	NA	8	None	ITT	68/213; 375/431; 164/218; 367/421	NA	15/213; 13/431; 10/218; 20/422
Bate[74]	1996	Europe	Placebo; Omeprazole 20mg QD	111(69); 98(56)	51±14; 47±14	SM: 0,1	NA	4	Omeprazole	ITT	NA	21/111; 56/98 *	22/111; 18/98
Robinson[75]	1995	USA	Ranitidine 150mg BID; Lansoprazole 30mg QD	127(48); 115(43)	NA	HD: 2-4	NA	8	None	ITT	83/127; 102/115	NA	4/127; 1/115
Mossner[76]	1995	Germany	Omeprazole 20mg QD; Pantoprazole 40mg QD	95(29); 191(58)	55(21-81); 53(19-89)	SM: 2,3	NA	8	None	PP	81/86;	81/86; 149/165	1/95; 2/191

											153/170		
Koop[77]	1995	USA	Ranitidine 150mg BID; Pantoprazole 40mg QD	83(17); 166(97)	53; 53	SM: 2,3	NA	8	None	ITT	46/83; 122/166	35/83; 104/166	NA
Bardhan[78]	1995	UK	Ranitidine 150mg BID; Lansoprazole 30mg QD; Lansoprazole 60mg QD	77(22); 77（27); 75(25)	49.3(19-74); 48(19-78); 47.9(18-74)	Reflux grades: 1-3	NA	8	None	ITT	37/77; 70/77; 59/75	30/77; 61/77; 54/75 *	20/77; 20/77; 16/75
Simon[79]	1993	USA	Ranitidine 150mg BID; Famotidine 20mg BID; Famotidine 40mg BID	172(65); 93(31); 175(61)	48.9; 52.2; 50.5	Modified SM: 2-4	NA	6	None	ITT	76/172; 48/93; 81/175	117/172; 56/93; 119/175 *	NA
Euler[80]	1993	USA	Placebo; Ranitidine 150mg QID; Ranitidine 300mg QID	116(34); 106(28); 106(23)	48.6±1.4; 46.3±1.5; 49.7±1.5	HD: 2-4	NA	8	Ranitidine	ITT	33/116; 73/106; 66/106	NA	37/116; 8/106; 12/106
Roufail[81]	1992	USA	Placebo; Ranitidine 150mg QID; Ranitidine 300mg QID	113(28); 109(34); 120(38)	48.6±1.3; 49.3±1.4; 48.4±1.3	HD: 2-4	NA	8	None	ITT	30/113; 68/109; 73/120	NA	33/113; 15/109; 18/120
Cloud[82]	1992	USA and Canada	Placebo; Nizatidine 150mg BID; Nizatidine 300mg BID	178(67); 168(81); 169(77)	44.78; 44.78; 45.83	Endoscopically Grade: 1-4	NA	6	None	ITT	46/178; 69/168; 65/169	NA	45/178; 19/168; 26/169
Sabesin[83]	1991	USA	Placebo; Famotidine 20mg BID; Famotidine 40mg QD	66(27); 137(63); 135(56)	46.9±15.9; 47.3±13.7; 47.9±14.6	HD: 0-4	NA	6	None	ITT	4/66; 47/137; 39/135	26/66; 88/137; 99/135	22/66; 33/137; 37/135
Robinson[84]	1991	USA	Placebo; Famotidine 20mg BID; Famotidine 40mg QD	76(31); 158(85); 155(81)	44.5±13.0; 45.2±14.2; 44.2±14.7	HD: 0,1	NA	6	Famotidine	ITT	NA	29/76; 95/158; 71/155 *	NA
Riemann[85]	1991	Germany	Placebo; Cimetidine 200mg QID	60(35); 60(26)	47.1(18-73); 48.9(18-69)	Heartburn: Mild	≥ 30 months	2	None	ITT	NA	12/65; 22/60	0/65; 0/60
Quik[86]	1990	Europe	Placebo; Nizatidine 300mg QD; Nizatidine 300mg BID	107(40); 109(40); 109(37)	52±17; 53±16; 53±16	NA	≥ 3 months	6	None	ITT	28/107; 33/109; 44/109	NA	10/107; 11/109; 7/109

Palmer[87]	1990	USA	Placebo; Cimetidine 800mg BID	86(28); 93(26)	51±15; 50±16	NA	NA	6	None	ITT	17/86; 47/93	NA	27/86; 16/93
Lundell[88]	1990	Sweden	Ranitidine 300mg BID; Omeprazole 40mg QD	47(11); 51(15)	59.8±16.0; 58.1±16.2	SM: 2-4	NA	8	None	ITT	18/47; 44/51	15/47; 44/51 *	NA
Dehn[89]	1990	UK	Cimetidine 400mg QID; Omeprazole 40mg QD	36(8); 31(10)	39.6(24-78); 54(21-74)	Endoscopically Grade: 2-4	NA	8	None	PP	7/31; 20/31	16/30; 23/31	5/36; 3/31
Vantrappen[90]	1988	Europe	Ranitidine 150mg BID; Omeprazole 40mg QD	30(21); 31(23)	52.3(24-79); 55.4(26-79)	Endoscopically Grade: 2,3	NA	8	None	ITT	15/30; 25/31	11/30; 27/31	5/30; 6/31
Havelund[91]	1988	Denmark	Ranitidine 150mg BID; Omeprazole 40mg QD	82(26); 80(32)	54.9(22-79); 53.7(24-79)	Endoscopically Grade: 1-3	NA	8	Ranitidine, Omeprazole	ITT	44/82; 69/80	NA	9/82; 5/80
Sontag[92]	1987	USA	Placebo; Ranitidine 150mg BID	118(42); 119(39)	48.2±1.40; 49.5±1.33	Endoscopy grade: Normal, Moderate, Severe	NA	6	Ranitidine	PP	29/71; 41/73	NA	32/150; 15/134

QD: Quaque die; BID: Bis in die; TID: Ter in die; QID: Quaque in die; A Chinese GerdQ reflux score[93]: A 20-item GERD questionnaire in the Chinese language; LA[94]: The Los Angeles grade; SM[95]: The Savary-Miller criteria; HD[96]: The Hetzel-Dent scale; ITT: Intention-to-treat data; PP: Per-protocol data; NA: Not available; *: Complete relief of symptoms.

References

1. Kim, E. H. *et al.* Efficacy of Lafutidine Versus Famotidine in Patients with Reflux Esophagitis: A Multi-Center, Randomized, Double-Blind, Non-inferiority Phase III Trial. *Dig Dis Sci*. **60**, 1724-1732, doi:10.1007/s10620-014-3489-4 [doi] (2015).
2. Peura, D. A. *et al.* Esomeprazole Treatment of Frequent Heartburn: Two Randomized, Double-Blind, Placebo-Controlled Trials. *Postgraduate Medicine*. **126**, 33-41, doi:10.3810/pgm.2014.07.2781 (2015).
3. Moraes-Filho, J. P. *et al.* Randomised clinical trial: daily pantoprazole magnesium 40 mg vs. esomeprazole 40 mg for gastro-oesophageal reflux disease, assessed by endoscopy and symptoms. *Aliment Pharmacol Ther*. **39**, 47-56, doi:10.1111/apt.12540 (2014).
4. Cho, Y. K. *et al.* Efficacy of pantoprazole 20 mg compared with pantoprazole 40 mg in the treatment of reflux esophagitis: a randomized, double-blind comparative trial. *Dig Dis Sci*. **57**, 3189-3194, doi:10.1007/s10620-012-2297-y (2012).
5. Kobeissy, A. A. *et al.* A randomized open-label trial of on-demand rabeprazole vs ranitidine for patients with non-erosive reflux disease. *World J Gastroenterol*. **18**, 2390-2395, doi:10.3748/wjg.v18.i19.2390 (2012).
6. Tan, V. P. *et al.* Treatment of non-erosive reflux disease with a proton pump inhibitor in Chinese patients: a randomized controlled trial. *J Gastroenterol*. **46**, 906-912, doi:10.1007/s00535-011-0402-1 (2011).
7. Laine, L. *et al.* Randomised clinical trial: a novel rabeprazole extended release 50 mg formulation vs. esomeprazole 40 mg in healing of moderate-to-severe erosive oesophagitis - the results of two double-blind studies. *Aliment Pharmacol Ther*. **33**, 203-212, doi:10.1111/j.1365-2036.2010.04516.x (2011).
8. Kinoshita, Y. *et al.* Randomised clinical trial: a multicentre, double-blind, placebo-controlled study on the efficacy and safety of rabeprazole 5 mg or 10 mg once daily in patients with non-erosive reflux disease. *Aliment Pharmacol Ther*. **33**, 213-224, doi:10.1111/j.1365-2036.2010.04508.x (2011).
9. Ohara, S. *et al.* A double-blind, controlled study comparing lafutidine with placebo and famotidine in Japanese patients with mild reflux esophagitis. *J Gastroenterol*. **45**, 1219-1227, doi:10.1007/s00535-010-0283-8 (2010).
10. Johnson, D. *et al.* Clinical trial: esomeprazole for moderate-to-severe nighttime heartburn and gastro-oesophageal reflux disease-related sleep disturbances. *Aliment Pharmacol Ther*. **32**, 182-190, doi:10.1111/j.1365-2036.2010.04339.x (2010).
11. Zheng, R.-N. Comparative study of omeprazole, lansoprazole, pantoprazole and esomeprazole for symptom relief in patients with reflux esophagitis. *World Journal of Gastroenterology*. **15**, 990, doi:10.3748/wjg.15.990 (2009).
12. Peura, D. A. *et al.* Clinical trial: lansoprazole 15 or 30 mg once daily vs. placebo for treatment of frequent nighttime heartburn in self-treating subjects. *Aliment Pharmacol Ther*. **30**, 459-468, doi:10.1111/j.1365-2036.2009.04064.x (2009).
13. Kushner, P. R. *et al.* Lansoprazole 15 mg once daily for 14 days is effective for treatment of frequent heartburn: results of 2 randomized, placebo-controlled,

double-blind studies. *Postgrad Med*. **121**, 67-75, doi:10.3810/pgm.2009.07.2019 (2009).

14. Eggleston, A. *et al.* Clinical trial: the treatment of gastro-oesophageal reflux disease in primary care--prospective randomized comparison of rabeprazole 20 mg with esomeprazole 20 and 40 mg. *Aliment Pharmacol Ther*. **29**, 967-978, doi:10.1111/j.1365-2036.2009.03948.x (2009).
15. Uemura, N. *et al.* Efficacy and safety of omeprazole in Japanese patients with nonerosive reflux disease. *J Gastroenterol*. **43**, 670-678, doi:10.1007/s00535-008-2214-5 (2008).
16. Hongo, M. *et al.* A randomized, double-blind, placebo-controlled clinical study of the histamine H2-receptor antagonist famotidine in Japanese patients with nonerosive reflux disease. *J Gastroenterol*. **43**, 448-456, doi:10.1007/s00535-008-2186-5 (2008).
17. Pilotto, A. *et al.* Comparison of four proton pump inhibitors for the short-term treatment of esophagitis in elderly patients. *World J Gastroenterol*. **13**, 4467-4472 (2007).
18. Monnikes, H. *et al.* Novel measurement of rapid treatment success with ReQuest: first and sustained symptom relief as outcome parameters in patients with endoscopy-negative GERD receiving 20 mg pantoprazole or 20 mg esomeprazole. *Digestion*. **75 Suppl 1**, 62-68, doi:10.1159/000101084 (2007).
19. Glatzel, D. *et al.* Pantoprazole 40 mg is as effective as esomeprazole 40 mg to relieve symptoms of gastroesophageal reflux disease after 4 weeks of treatment and superior regarding the prevention of symptomatic relapse. *Digestion*. **75 Suppl 1**, 69-78, doi:10.1159/000101085 (2007).
20. Bardhan, K. D. *et al.* A clinical trial comparing pantoprazole and esomeprazole to explore the concept of achieving 'complete remission' in gastro-oesophageal reflux disease. *Aliment Pharmacol Ther*. **25**, 1461-1469, doi:10.1111/j.1365-2036.2007.03337.x (2007).
21. Bytzer, P. *et al.* Effect of rabeprazole and omeprazole on the onset of gastro-oesophageal reflux disease symptom relief during the first seven days of treatment. *Scand J Gastroenterol*. **41**, 1132-1140, doi:10.1080/00365520600615781 (2006).
22. Lightdale, C. J. *et al.* A multicenter, randomized, double-blind, 8-week comparative trial of low-dose esomeprazole (20 mg) and standard-dose omeprazole (20 mg) in patients with erosive esophagitis. *Dig Dis Sci*. **51**, 852-857, doi:10.1007/s10620-005-9071-3 (2006).
23. Schmitt, C. *et al.* A multicenter, randomized, double-blind, 8-week comparative trial of standard doses of esomeprazole (40 mg) and omeprazole (20 mg) for the treatment of erosive esophagitis. *Dig Dis Sci*. **51**, 844-850, doi:10.1007/s10620-005-9062-4 (2006).
24. Pace, F. *et al.* Rabeprazole is equivalent to omeprazole in the treatment of erosive gastro-oesophageal reflux disease. A randomised, double-blind, comparative study of rabeprazole and omeprazole 20 mg in acute treatment of reflux oesophagitis, followed by a maintenance open-label, low-dose therapy with rabeprazole. *Dig Liver Dis*. **37**, 741-750, doi:10.1016/j.dld.2005.04.026 (2005).
25. Kahrilas, P. J. *et al.* Efficacy of rabeprazole in the treatment of symptomatic gastroesophageal reflux disease. *Dig Dis Sci*. **50**, 2009-2018, doi:10.1007/s10620-005-3000-3 (2005).

26. Johnson, D. A. *et al.* Effect of esomeprazole on nighttime heartburn and sleep quality in patients with GERD: a randomized, placebo-controlled trial. *Am J Gastroenterol*. **100**, 1914-1922, doi:10.1111/j.1572-0241.2005.00285.x (2005).
27. Fock, K. M. *et al.* Rabeprazole vs esomeprazole in non-erosive gastro-esophageal reflux disease: a randomized, double-blind study in urban Asia. *World J Gastroenterol*. **11**, 3091-3098 (2005).
28. Fennerty, M. B. *et al.* Efficacy of esomeprazole 40 mg vs. lansoprazole 30 mg for healing moderate to severe erosive oesophagitis. *Aliment Pharmacol Ther*. **21**, 455-463, doi:10.1111/j.1365-2036.2005.02339.x (2005).
29. van Zyl, J. *et al.* Efficacy and safety of pantoprazole versus ranitidine in the treatment of patients with symptomatic gastroesophageal reflux disease. *Digestion*. **70**, 61-69, doi:10.1159/000080130 (2004).
30. Gillessen, A. *et al.* 40 mg pantoprazole and 40 mg esomeprazole are equivalent in the healing of esophageal lesions and relief from gastroesophageal reflux disease-related symptoms. *J Clin Gastroenterol*. **38**, 332-340 (2004).
31. Armstrong, D. *et al.* The role of acid suppression in patients with endoscopy-negative reflux disease: the effect of treatment with esomeprazole or omeprazole. *Aliment Pharmacol Ther*. **20**, 413-421, doi:10.1111/j.1365-2036.2004.02085.x (2004).
32. Scholten, T. *et al.* Once-daily pantoprazole 40 mg and esomeprazole 40 mg have equivalent overall efficacy in relieving GERD-related symptoms. *Alimentary Pharmacology and Therapeutics*. **18**, 587-594, doi:10.1046/j.1365-2036.2003.01745.x (2003).
33. Katz, P. O. *et al.* Esomeprazole resolves chronic heartburn in patients without erosive oesophagitis. *Aliment Pharmacol Ther*. **18**, 875-882 (2003).
34. Korner, T. *et al.* Comparable efficacy of pantoprazole and omeprazole in patients with moderate to severe reflux esophagitis. Results of a multinational study. *Digestion*. **67**, 6-13, doi:70201 (2003).
35. Kao, A. W. *et al.* On-demand therapy for Los Angeles grade A and B reflux esophagitis: esomeprazole versus omeprazole. *J Formos Med Assoc*. **102**, 607-612 (2003).
36. Adachi, K. *et al.* Symptom relief in patients with reflux esophagitis: comparative study of omeprazole, lansoprazole, and rabeprazole. *J Gastroenterol Hepatol*. **18**, 1392-1398 (2003).
37. Mulder, C. J. *et al.* A double-blind, randomized comparison of omeprazole Multiple Unit Pellet System (MUPS) 20 mg, lansoprazole 30 mg and pantoprazole 40 mg in symptomatic reflux oesophagitis followed by 3 months of omeprazole MUPS maintenance treatment: a Dutch multicentre trial. *Eur J Gastroenterol Hepatol*. **14**, 649-656 (2002).
38. Miner, P., Jr. *et al.* Rabeprazole in nonerosive gastroesophageal reflux disease: a randomized placebo-controlled trial. *Am J Gastroenterol*. **97**, 1332-1339, doi:10.1111/j.1572-0241.2002.05769.x (2002).

39. Meneghelli, U. G. *et al.* Efficacy and tolerability of pantoprazole versus ranitidine in the treatment of reflux esophagitis and the influence of Helicobacter pylori infection on healing rate. *Dis Esophagus*. **15**, 50-56 (2002).
40. Kovacs, T. O. *et al.* Comparison of the efficacy of pantoprazole vs. nizatidine in the treatment of erosive oesophagitis: a randomized, active-controlled, double-blind study. *Aliment Pharmacol Ther*. **16**, 2043-2052 (2002).
41. Holtmann, G. *et al.* A randomized, double-blind, comparative study of standard-dose rabeprazole and high-dose omeprazole in gastro-oesophageal reflux disease. *Aliment Pharmacol Ther*. **16**, 479-485 (2002).
42. Castell, D. O. *et al.* Esomeprazole (40 mg) compared with lansoprazole (30 mg) in the treatment of erosive esophagitis. *Am J Gastroenterol*. **97**, 575-583, doi:10.1111/j.1572-0241.2002.05532.x (2002).
43. Richter, J. E. *et al.* Comparing lansoprazole and omeprazole in onset of heartburn relief: results of a randomized, controlled trial in erosive esophagitis patients. *Am J Gastroenterol*. **96**, 3089-3098, doi:10.1111/j.1572-0241.2001.05263.x (2001).
44. Richter, J. E. *et al.* Efficacy and safety of esomeprazole compared with omeprazole in GERD patients with erosive esophagitis: a randomized controlled trial. *Am J Gastroenterol*. **96**, 656-665, doi:10.1111/j.1572-0241.2001.3600_b.x (2001).
45. Paul, K. *et al.* Effectiveness and safety of nizatidine, 75 mg, for the relief of episodic heartburn. *Aliment Pharmacol Ther*. **15**, 1571-1577 (2001).
46. Kaspari, S. *et al.* Comparison of pantoprazole 20 mg to ranitidine 150 mg b.i.d. in the treatment of mild gastroesophageal reflux disease. *Digestion*. **63**, 163-170, doi:51885 (2001).
47. Bardhan, K. D. *et al.* Comparable clinical efficacy and tolerability of 20 mg pantoprazole and 20 mg omeprazole in patients with grade I reflux oesophagitis. *Aliment Pharmacol Ther*. **15**, 1585-1591 (2001).
48. Armstrong, D. *et al.* Symptom relief in gastroesophageal reflux disease: a randomized, controlled comparison of pantoprazole and nizatidine in a mixed patient population with erosive esophagitis or endoscopy-negative reflux disease. *Am J Gastroenterol*. **96**, 2849-2857, doi:10.1111/j.1572-0241.2001.4237_a.x (2001).
49. van Zyl, J. H. *et al.* Efficacy and tolerability of 20 mg pantoprazole versus 300 mg ranitidine in patients with mild reflux-oesophagitis: a randomized, double-blind, parallel, and multicentre study. *Eur J Gastroenterol Hepatol*. **12**, 197-202 (2000).
50. Richter, J. E. *et al.* Efficacy of omeprazole for the treatment of symptomatic acid reflux disease without esophagitis. *Arch Intern Med*. **160**, 1810-1816 (2000).
51. Richter, J. E. *et al.* Oral pantoprazole for erosive esophagitis: a placebo-controlled, randomized clinical trial. Pantoprazole US GERD Study Group. *Am J Gastroenterol*. **95**, 3071-3080, doi:10.1111/j.1572-0241.2000.03254.x (2000).
52. Kahrilas, P. J. *et al.* Esomeprazole improves healing and symptom resolution as compared with omeprazole in reflux oesophagitis patients: a randomized controlled trial. The Esomeprazole Study Investigators. *Aliment Pharmacol Ther*. **14**, 1249-1258 (2000).

53. Fass, R. *et al.* Omeprazole 40 mg once a day is equally effective as lansoprazole 30 mg twice a day in symptom control of patients with gastro-oesophageal reflux disease (GERD) who are resistant to conventional-dose lansoprazole therapy-a prospective, randomized, multi-centre study. *Aliment Pharmacol Ther*. **14**, 1595-1603 (2000).
54. Farley, A. *et al.* Rabeprazole versus ranitidine for the treatment of erosive gastroesophageal reflux disease: a double-blind, randomized clinical trial. Raberprazole Study Group. *Am J Gastroenterol*. **95**, 1894-1899, doi:10.1111/j.1572-0241.2000.02233.x (2000).
55. Delchier, J. C. *et al.* Rabeprazole, 20 mg once daily or 10 mg twice daily, is equivalent to omeprazole, 20 mg once daily, in the healing of erosive gastrooesophageal reflux disease. *Scand J Gastroenterol*. **35**, 1245-1250 (2000).
56. Richter, J. E. *et al.* Lansoprazole in the treatment of heartburn in patients without erosive oesophagitis. *Aliment Pharmacol Ther*. **13**, 795-804 (1999).
57. Pappa, K. A. *et al.* Low-dose ranitidine for the relief of heartburn. *Aliment Pharmacol Ther*. **13**, 459-465 (1999).
58. Pappa, K. A. *et al.* An evaluation of increasing doses of ranitidine for treatment of heartburn. *Aliment Pharmacol Ther*. **13**, 475-481 (1999).
59. Maton, P. N. *et al.* Efficacy of omeprazole versus ranitidine for symptomatic treatment of poorly responsive acid reflux disease-a prospective, controlled trial. *Aliment Pharmacol Ther*. **13**, 819-826 (1999).
60. Jones, R. *et al.* Low-dose lansoprazole provides greater relief of heartburn and epigastric pain than low-dose omeprazole in patients with acid-related dyspepsia. *Aliment Pharmacol Ther*. **13**, 413-419 (1999).
61. Jansen, J. B. *et al.* Standard-dose lansoprazole is more effective than high-dose ranitidine in achieving endoscopic healing and symptom relief in patients with moderately severe reflux oesophagitis. The Dutch Lansoprazole Study Group. *Aliment Pharmacol Ther*. **13**, 1611-1620 (1999).
62. Festen, H. P. *et al.* Omeprazole versus high-dose ranitidine in mild gastroesophageal reflux disease: short- and long-term treatment. The Dutch Reflux Study Group. *Am J Gastroenterol*. **94**, 931-936, doi:10.1111/j.1572-0241.1999.989_l.x (1999).
63. Galmiche, J. P. *et al.* On-demand treatment of gastro-oesophageal reflux symptoms: a comparison of ranitidine 75 mg with cimetidine 200 mg or placebo. *Aliment Pharmacol Ther*. **12**, 909-917 (1998).
64. Earnest, D. L. *et al.* A placebo-controlled dose-ranging study of lansoprazole in the management of reflux esophagitis. *Am J Gastroenterol*. **93**, 238-243, doi:10.1111/j.1572-0241.1998.00238.x (1998).
65. Dettmer, A. *et al.* Pantoprazole 20 mg is effective for relief of symptoms and healing of lesions in mild reflux oesophagitis. *Aliment Pharmacol Ther*. **12**, 865-872 (1998).
66. Venables, T. L. *et al.* Omeprazole 10 milligrams once daily, omeprazole 20 milligrams once daily, or ranitidine 150 milligrams twice daily, evaluated as initial therapy for the relief of symptoms of gastro-oesophageal reflux disease in general practice. *Scand J Gastroenterol*. **32**, 965-973, doi:10.3109/00365529709011211

(1997).

67. Galmiche, J. P. *et al.* Treating the symptoms of gastro-oesophageal reflux disease: a double-blind comparison of omeprazole and cisapride. *Aliment Pharmacol Ther*. **11**, 765-773 (1997).

68. Bate, C. M. *et al.* Omeprazole is more effective than cimetidine for the relief of all grades of gastro-oesophageal reflux disease-associated heartburn, irrespective of the presence or absence of endoscopic oesophagitis. *Aliment Pharmacol Ther*. **11**, 755-763 (1997).

69. Silver, M. T. *et al.* Ranitidine 300 mg twice daily and 150 mg four-times daily are effective in healing erosive oesophagitis. *Aliment Pharmacol Ther*. **10**, 373-380 (1996).

70. van Rensburg, C. J. *et al.* Efficacy and tolerability of pantoprazole 40 mg versus 80 mg in patients with reflux oesophagitis. *Aliment Pharmacol Ther*. **10**, 397-401 (1996).

71. Mee, A. S. *et al.* Rapid symptom relief in reflux oesophagitis: a comparison of lansoprazole and omeprazole. *Aliment Pharmacol Ther*. **10**, 757-763 (1996).

72. McCarty-Dawson, D. *et al.* Ranitidine versus cimetidine in the healing of erosive esophagitis. *Clin Ther*. **18**, 1150-1160 (1996).

73. Castell, D. O. *et al.* Efficacy and safety of lansoprazole in the treatment of erosive reflux esophagitis. The Lansoprazole Group. *Am J Gastroenterol*. **91**, 1749-1757 (1996).

74. Bate, C. M. *et al.* Reflux symptom relief with omeprazole in patients without unequivocal oesophagitis. *Aliment Pharmacol Ther*. **10**, 547-555 (1996).

75. Robinson, M. *et al.* A comparison of lansoprazole and ranitidine in the treatment of erosive oesophagitis. Multicentre Investigational Group. *Aliment Pharmacol Ther*. **9**, 25-31 (1995).

76. Mossner, J. *et al.* A double-blind study of pantoprazole and omeprazole in the treatment of reflux oesophagitis: a multicentre trial. *Aliment Pharmacol Ther*. **9**, 321-326 (1995).

77. Koop, H. *et al.* Comparative trial of pantoprazole and ranitidine in the treatment of reflux esophagitis. Results of a German multicenter study. *J Clin Gastroenterol*. **20**, 192-195 (1995).

78. Bardhan, K. D. *et al.* Lansoprazole versus ranitidine for the treatment of reflux oesophagitis. UK Lansoprazole Clinical Research Group. *Aliment Pharmacol Ther*. **9**, 145-151 (1995).

79. Simon, T. J. *et al.* Efficacy of twice daily doses of 40 or 20 milligrams famotidine or 150 milligrams ranitidine for treatment of patients with moderate to severe erosive esophagitis. Famotidine Erosive Esophagitis Study Group. *Scand J Gastroenterol*. **28**, 375-380 (1993).

80. Euler, A. R. *et al.* Ranitidine is effective therapy for erosive esophagitis. *Am J Gastroenterol*. **88**, 520-524 (1993).

81. Roufail, W. *et al.* Ranitidine for erosive oesophagitis: a double-blind, placebo-controlled study. Glaxo Erosive Esophagitis Study Group. *Aliment Pharmacol Ther*. **6**,

597-607 (1992).

82. Cloud, M. L. *et al.* Nizatidine versus placebo in gastroesophageal reflux disease. A six-week, multicenter, randomized, double-blind comparison. Nizatidine Gastroesophageal Reflux Disease Study Group. *Dig Dis Sci*. **37**, 865-874 (1992).

83. Sabesin, S. M. *et al.* Famotidine relieves symptoms of gastroesophageal reflux disease and heals erosions and ulcerations. Results of a multicenter, placebo-controlled, dose-ranging study. USA Merck Gastroesophageal Reflux Disease Study Group. *Arch Intern Med*. **151**, 2394-2400 (1991).

84. Robinsen, M. *et al.* Famotidine (20 mg) b.d. relieves gastrooesophageal reflux symptoms in patients without erosive oesophagitis. Famotidine/GERD Investigation Group. *Aliment Pharmacol Ther*. **5**, 631-643 (1991).

85. Riemann, J. F. *et al.* Cimetidine suspension in patients with stage 0 gastro-oesophageal reflux disease. *Aliment Pharmacol Ther*. **5**, 191-197 (1991).

86. Quik, R. F. *et al.* A comparison of two doses of nizatidine versus placebo in the treatment of reflux oesophagitis. *Aliment Pharmacol Ther*. **4**, 201-211 (1990).

87. Palmer, R. H. *et al.* Cimetidine 800 mg twice daily for healing erosions and ulcers in gastroesophageal reflux disease. *J Clin Gastroenterol*. **12 Suppl 2**, S29-34 (1990).

88. Lundell, L. *et al.* Omeprazole or high-dose ranitidine in the treatment of patients with reflux oesophagitis not responding to 'standard doses' of H2-receptor antagonists. *Aliment Pharmacol Ther*. **4**, 145-155 (1990).

89. Dehn, T. C. *et al.* Double blind comparison of omeprazole (40 mg od) versus cimetidine (400 mg qd) in the treatment of symptomatic erosive reflux oesophagitis, assessed endoscopically, histologically and by 24 h pH monitoring. *Gut*. **31**, 509-513 (1990).

90. Vantrappen, G. *et al.* Omeprazole (40 mg) is superior to ranitidine in short-term treatment of ulcerative reflux esophagitis. *Dig Dis Sci*. **33**, 523-529 (1988).

91. Havelund, T. *et al.* Omeprazole and ranitidine in treatment of reflux oesophagitis: double blind comparative trial. *Br Med J (Clin Res Ed)*. **296**, 89-92 (1988).

92. Sontag, S. *et al.* Ranitidine therapy for gastroesophageal reflux disease. Results of a large double-blind trial. *Arch Intern Med*. **147**, 1485-1491 (1987).

93. Wong, W. M. *et al.* A validated symptoms questionnaire (Chinese GERDQ) for the diagnosis of gastro-oesophageal reflux disease in the Chinese population. *Aliment Pharmacol Ther*. **17**, 1407-1413 (2003).

94. Lundell, L. R. *et al.* Endoscopic assessment of oesophagitis: clinical and functional correlates and further validation of the Los Angeles classification. *Gut*. **45**, 172-180 (1999).

95. Ollyo., J. *et al.* Savary-Miller's new endoscopic grading of reflux-oesophagitis: a simple, reproducible, logical, complete and useful classification. *Gastroenterology*. **98**, A-100 (1990).

96. Hetzel, D. J. *et al.* Healing and relapse of severe peptic esophagitis after treatment with omeprazole. *Gastroenterology*. **95**, 903-912 (1988).

Supplementary Table S2 Adjusting the confounding factors in all outcomes

Confounding factors	Healing	Relief of symptoms	Tolerance
**Area**			
Europe	Ref	Ref	Ref
North America	0.674 (-0.324, 1.031)	-0.101(-0.886, 0.635)	-0.02(-0.371, 0.319)
Asia	1.244(-0.875, 3.954)	-0.448(-1.390, 0.432)	0.091(-0.500, 0.672)
Other/mix	-12.87(-66.25, 25.47)	-2.801(-3.157, 12.10)	-1.398(-20.7, 15.32)
σ	0.832	0.469	0.084
**Gender**			
Coefficient	-0.127(-0.225, -0.026)	-0.014(-0.036, 0.009)	0.010(-0.004, 0.021)
σ	0.941	0.462	0.076
**Mean age**			
Coefficient	-0.130(-0.372, 0.107)	0.029(-0.067, 0.131)	0.001(-0.060, 0.058)
σ	0.996	0.469	0.080
**Level of disease from endoscopy**			
Coefficient	-0.348(-4.953, 4.284)	NA	-0.006(-0.015, 0.005)
σ	1.013	NA	0.090
**Level of disease from symptoms**			
Coefficient	NA	1.275(-1.144, 3.741)	-0.008(-0.041, 0.240)
σ	NA	0.467	0.079
**Degree of relieve symptoms**			
Complete relief	NA	Ref	NA
Relief	NA	0.012(-0.517, 0.543)	NA
σ	NA	0.471	NA
**Methodological quality score**			
Coefficient	-1.006(-1.808, -0.213)	-0.048(-0.233, 0.141)	0.085(-0.049, 0.218)
σ	0.943	0.469	0.081
**Year of publication**			
Coefficient	-0.046(-0.156, 0.062)	-0.051(-0.088, 0.012)	0.028(0.008, 0.049)
σ	1.005	0.433	0.064
**Pharmaceutical industry sponsorship**			
None	Ref	Ref	Ref
Sponsorship	0.811(-0.341, 1.955)	-0.240(-0.808, 0.353)	0.100(-0.215, 0.408)
σ	0.990	0.464	0.075
**ITT/PP**			
ITT	Ref	Ref	Ref
PP	1.249(-0.063, 2.565)	-0.698(-1.847, 0.460)	-0.087(-0.626, 0.440)
σ	0.972	0.464	0.076
**Withdrawal rate**			
Coefficient	-0.023(-0.002, 0.119)	0.021(-0.022, 0.064)	NA
σ	1.008	0.465	NA

Coefficient of posterior median, and 95% credible interval (CrIs) of the between-trial heterogeneity (σ) in ln(OR) scale for all outcomes using the random effects models; Ref, Reference; NA, Not available. If the coefficient was positive value, the effect was expanded by

corresponding confounding factor in corresponding outcome; Otherwise, the opposite.

Supplementary Table S3 Adjusting selective design biases and model parameters in all outcomes

Model	Healing	Relief of symptoms	Tolerance
**Model: No adjustment**			
PD	98.7	139.8	107.9
D	117.1	162.3	172.7
DIC	215.8	302.1	280.6
σ	0.383	0.461	0.077
**Model 1.1: New favored - Fixed coefficient**			
Coefficient	0.42(-0.91, 1.78)	-0.17(-1.24, 0.90)	-0.02(-0.49, 0.43)
PD	96.6	140.5	108.9
D	117.6	162.3	174.5
DIC	214.2	302.8	283.4
σ	0.327	0.464	0.078
**Model 1.2: New favored - Exchangeable coefficients**			
Coefficient	1.40(-3.00, 5.84)	4.27(-2.84, 11.2)	-0.21(-1.36, 0.96)
PD	96.3	140.2	109.5
D	118.0	162.4	174.1
DIC	214.3	302.6	283.6
σ	0.317	0.460	0.082
**Model 1.3: New favored - Consistent coefficients**			
PD	104.2	143.3	129.2
D	121.0	161.0	184.0
DIC	225.2	304.3	313.2
σ	0.314	0.428	0.093
**Model 2.1: Sponsored favored - Fixed coefficient**			
Coefficient	0.72(-2.84, 4.21)	-0.74(-5.39, 4.20)	-0.21(-1.36, 0.96)
PD	96.4	140.4	109.3
D	117.9	162.4	173.9
DIC	214.3	302.8	283.2
σ	0.326	0.466	0.081
**Model 2.2: Sponsored favored - Exchangeable coefficients**			
Coefficient	-2.12(-6.38, 2.33)	0.87(-4.92, 7.53)	0.19(-1.02, 1.47)
PD	96.6	140.7	110.7
D	117.7	162.3	175.2
DIC	214.3	303.0	285.9
σ	0.324	0.467	0.081
**Model 2.3: Sponsored favored - Consistent coefficients**			
PD	101.1	144.3	123.8
D	117.0	161.5	178.8
DIC	218.1	305.8	302.6
σ	0.318	0.503	0.085
**Model 3.1: High-dose favored - Fixed coefficient**			
Coefficient	-1.06(-2.79, 0.67)	0.37(-3.84, 4.79)	0.51(-0.60, 1.60)
PD	96.3	140.3	109.7

D	117.9	162.0	173.3
DIC	214.2	302.3	283.0
σ	0.321	0.464	0.084
**Model 3.2: High-dose favored - Exchangeable coefficients**			
Coefficient	-1.33(-6.40, 3.32)	-0.33(-4.94, 4.28)	0.25(-1.04, 1.69)
PD	96.7	140.4	110.1
D	117.4	162.4	174.1
DIC	214.1	302.8	284.2
σ	0.327	0.465	0.080
**Model 3.3: High-dose favored - Consistent coefficients**			
PD	102.4	142.0	119.2
D	118.8	161.2	179.3
DIC	220.2	303.2	298.5
σ	0.322	0.475	0.084
**Model 4.1: PPIs favored - Fixed coefficient**			
Coefficient	1.14(-1.36, 3.64)	4.49(1.26, 7.85)	0.35(-0.72, 1.45)
PD	96.2	138.3	109.7
D	118.1	163.1	174.0
DIC	214.3	301.4	283.7
σ	0.323	0.422	0.083
**Model 4.2: PPIs favored - Exchangeable coefficients**			
Coefficient	1.98(-3.10, 6.98)	2.29(1.32, 7.55)	0.41(-0.71, 1.62)
PD	96.1	139.8	108.3
D	118.6	162.1	173.6
DIC	214.7	301.9	281.9
σ	0.316	0.459	0.078
**Model 4.3: PPIs favored - Consistent coefficients**			
PD	100.9	140.3	111.8
D	118.7	162.8	174.6
DIC	219.6	303.1	286.4
σ	0.302	0.442	0.080
**Model 5.1: Supplementary merger favored - Fixed coefficient**			
Coefficient	1.14(-1.36, 3.64)	3.98(-1.04, 6.89)	0.10(-0.67, 0.89)
PD	96.2	138.3	109.7
D	118.1	163.6	174.6
DIC	214.3	301.9	284.3
σ	0323	0.421	0.083
**Model 5.2: Supplementary merger favored - Exchangeable coefficients**			
Coefficient	1.98(-3.10, 6.98)	0.87(-4.92, 7.53)	0.19(-1.00, 1.47)
PD	96.1	140.7	110.7
D	118.6	162.3	175.2
DIC	214.7	303.0	285.9
σ	0.316	0.467	0.08
**Model 5.3: Supplementary merger favored - Consistent coefficients**			

PD	100.9	143.4	124.3
D	118.7	162.3	181.0
DIC	219.6	305.7	305.3
σ	0.302	0.470	0.097

Medians and 95% credible intervals from the posterior distributions of the heterogeneity (σ), two small-study effects coefficients in ln(OR) scale for all outcomes. Deviance information criterion (DIC) and posterior mean of the residual deviance (D) are also reported. Model 5 provide different merger favored in different outcomes, sponsored favored and high-dose favored for the healing rate, PPIs favored and sponsored favored for relief of symptoms, sponsored and PPIs favored for tolerance. If coefficient is found positive, it means that there are small-study effects in the direction of design bias, otherwise the opposite.

Supplementary Table S4 The results of compare models in all outcomes

	Healing				Relief of symptoms			Tolerance		
	**NM0**	**HM1**	**HM2**	**HM3**	**NM0**	**HM1**	**HM4**	**NM0**	**HM1**	**HM5**
PD	98.7	91.7	90.1	92.0	139.8	132.1	132.7	107.9	91.6	89.5
D	117.1	117.0	116.6	116.7	162.3	165.1	165.2	172.7	171.3	164.2
DIC	215.8	208.7	206.7	208.7	302.1	297.2	297.9	280.6	262.9	253.7
σ	0.383	0.334	0.306	0.332	0.461	0.418	0.410*; 0.107#	0.077	0.066	0.055

NM0: No-adjustment model;

HM1: The hierarchical modeling approach incorporating dose-related and PPI favored constraints;

HM2: The hierarchical modeling approach incorporating dose-related and PPI favored constraints with gender;

HM3: The hierarchical modeling approach incorporating dose-related and PPI favored constraints with methodological quality score;

HM4: The hierarchical modeling approach incorporating dose-related and PPI favored constraints with inconsistency;

HM5: The hierarchical modeling approach incorporating dose-related and PPI favored constraints with year of publication.

PD: number of parameters, D: posterior mean of the residual deviance, DIC: deviance information criterion, σ: Heterogeneity/Inconsistency, * indicates heterogeneity, # indicates inconsistency.

Supplementary Table S8 The baseline results of network analysis in sensitivity analyses

Treatment		Outcomes	Base case	SUCRA rank	Excluded single blinded and unblinded studies	SUCRA rank	Excluded study from China	SUCRA rank	Excluded "0" study	SUCRA rank	Excluded inconsistent node	SUCRA rank	Excluding the discontinued drug (cimetidine)	SUCRA rank
**Esomeprazole**	**20mg/day**	**Healing**	11.67(7.33, 17.66)	8	11.51(6.92, 15.9)	8	10.23(7.70, 16.1)	8	NA	NA	11.76(7.51, 13.8)	9	10.3(7.69,14.8)	8
		**Relief of symptoms**	3.86(2.86, 4.93)	10	3.93(2.91, 5.07)	10	NA	NA	NA	NA	NA	NA	3.81(2.84, 4.74)	10
		**Tolerance**	0.66(0.55, 0.81)	16	0.66(0.57, 0.75)	16	0.67(0.57, 0.79)	16	0.65(0.57, 0.79)	15	0.64(0.52, 0.76)	13	0.65(0.56, 0.77)	14
	**40mg/day**	**Healing**	15.45(10.24, 21.96)	1	14.91(9.02, 18.40)	1	14.21(9.85, 19.25)	1	NA	NA	14.8(8.38, 17.81)	1	15.1(9.82,19.57)	1
		**Relief of symptoms**	4.42(3.33, 5.77)	6	4.46(3.41, 5.90)	5	NA	NA	NA	NA	NA	NA	4.24(3.18, 5.52)	5
		**Tolerance**	0.61(0.51, 0.73)	7	0.62(0.57, 0.71)	6	0.63(0.58, 0.74)	5	0.61(0.51, 0.71)	5	0.63(0.50, 0.74)	6	0.63(0.54, 0.72)	5
**Lansoprazole**	**15mg/day**	**Healing**	8.23(4.87, 12.79)	12	8.81(4.71, 11.81)	12	6.82(4.17, 11.51)	12	NA	NA	NA	NA	7.91(4.45,10.8)	12
		**Relief of symptoms**	3.83(2.62, 5.21)	11	3.78(2.51, 5.17)	11	NA	NA	NA	NA	NA	NA	3.76(2.62, 5.01)	11
		**Tolerance**	0.68(0.55, 0.83)	19	0.68(0.53, 0.80)	18	0.68(0.60, 0.82)	18	0.65(0.54, 0.79)	19	0.65(0.57, 0.81)	19	0.68(0.57, 0.79)	15
	**30mg/day**	**Healing**	12.31(8.60, 17.03)	6	11.83(7.69, 16.17)	6	11.59(7.68, 17.23)	6	NA	NA	11.45(7.41, 16.48)	8	11.55(6.80,15.13)	6
		**Relief of symptoms**	4.54(3.30, 6.16)	5	4.32(3.34, 5.98)	7	NA	NA	NA	NA	NA	NA	4.34(3.27, 6.18)	6
		**Tolerance**	0.63(0.53, 0.75)	10	0.67(0.54, 0.73)	10	0.64(0.59, 0.71)	10	0.63(0.57, 0.70)	9	0.66(0.54, 0.71)	9	0.62(0.55, 0.70)	7
	**60mg/day**	**Healing**	14.10(9.44, 21.09)	5	13.91(9.11, 20.8)	5	13.7(9.74, 20.10)	5	NA	NA	13.41(8.67, 19.54)	5	15.20(8.78, 20.20)	4
		**Relief of symptoms**	5.19(3.59, 8.02)	2	4.98(3.78, 7.97)	3	NA	NA	NA	NA	NA	NA	4.82(3.71, 7.65)	2
		**Tolerance**	0.58(0.45, 0.71)	3	0.60(0.50, 0.70)	3	0.61(0.51, 0.69)	3	0.58(0.51, 0.71)	2	0.60(0.47, 0.70)	2	0.61(0.52, 0.69)	2
**Omeprazole**	**10mg/day**	**Healing**	NA	NA	NA	NA	NA	NA	NA	NA	NA	NA	NA	NA

		**Relief of symptoms**	3.42(2.31, 4.65)	14	3.40(2.37, 4.71)	14	NA	NA	NA	NA	NA	NA	3.37(2.17, 4.77)	14
		**Tolerance**	0.66(0.54, 0.83)	15	0.67(0.55, 0.81)	15	0.67(0.59, 0.81)	15	0.65(0.52, 0.80)	16	0.67(0.58, 0.74)	15	0.66(0.54, 0.78)	12
	**20mg/day**	**Healing**	10.18(6.93, 14.48)	10	10.02(5.96,13.84)	10	9.87(5.92, 13.91)	10	NA	NA	9.51(6.04, 12.54)	11	11.41(5.81,13.87)	10
		**Relief of symptoms**	4.21(3.22, 5.46)	7	4.31(3.37, 5.63)	8	NA	NA	NA	NA	NA	NA	4.17(3.19, 5.47)	7
		**Tolerance**	0.61(0.51, 0.72)	6	0.62(0.54, 0.71)	7	0.63(0.59, 0.72)	6	0.62(0.57, 0.73)	6	0.62(0.51, 0.70)	5	0.63(0.53, 0.71)	6
	**40mg/day**	**Healing**	14.93(9.56, 23.12)	3	13.73(9.48, 21.46)	4	13.77(8.56, 22.9)	4	NA	NA	11.69(8.2141, 23.4)	4	13.71(8.65, 24.6)	5
		**Relief of symptoms**	5.70(3.74, 9.52)	1	5.72(3.91, 9.78)	1	NA	NA	NA	NA	NA	NA	5.54(3.58, 8.43)	1
		**Tolerance**	0.57(0.44, 0.69)	1	0.57(0.52, 0.70)	1	0.55(0.53, 0.68)	1	0.57(0.47, 0.70)	1	0.60(0.46, 0.67)	1	0.62(0.51, 0.71)	1
**Pantoprazole**	**10-20mg/day**	**Healing**	6.88(4.23, 10.82)	13	6.84(4.32, 9.75)	13	5.78(4.32, 9.57)	13	NA	NA	5.96(2.89, 8.73)	12	5.23(3.79, 8.95)	13
		**Relief of symptoms**	3.54(2.40, 4.76)	13	3.58(2.41, 4.89)	12	NA	NA	NA	NA	NA	NA	3.43(2.43, 4.98)	13
		**Tolerance**	0.64(0.53, 0.78)	12	0.65(0.59, 0.76)	12	0.67(0.60, 0.78)	12	0.64(0.58, 0.77)	12	0.65(0.52, 0.76)	11	0.65(0.59, 0.73)	10
	**40mg/day**	**Healing**	11.64(8.16, 16.05)	9	10.20(7.98, 15.7)	9	11.77(6.91, 17.31)	9	NA	NA	10.96(7.65, 16.97)	7	9.98(6.78,15.20)	9
		**Relief of symptoms**	4.19(3.10, 5.58)	8	4.32(3.27, 6.04)	6	NA	NA	NA	NA	NA	NA	4.14(3.24, 5.63)	8
		**Tolerance**	0.59(0.48, 0.70)	2	0.60(0.51, 0.71)	2	0.61(0.52, 0.70)	2	0.60(0.51, 0.73)	3	0.58(0.50, 0.70)	3	0.61(0.54, 0.69)	3
	**80mg/day**	**Healing**	14.74(9.42, 23.30)	4	14.31(8.29, 21.91)	3	13.86(9.33, 21.70)	3	NA	NA	13.54(9.14, 20.15)	3	12.21(7.82,19.7)	3
		**Relief of symptoms**	4.95(3.43, 7.71)	3	5.14(3.67, 7.73)	2	NA	NA	NA	NA	NA	NA	4.64(3.51, 7.79)	3
		**Tolerance**	NA	NA	NA	NA	NA	NA	NA	NA	NA	NA	NA	NA
**Rabeprazole**	**5-10mg/day**	**Healing**	9.56(5.63, 14.62)	11	9.54(5.31, 13.12)	11	8.97(5.32, 12.84)	11	NA	NA	8.63(3.54, 12.91)	10	8.49(4.32,12.97)	11
		**Relief of symptoms**	3.61(2.50, 4.83)	12	3.47(2.64, 4.74)	13	NA	NA	NA	NA	NA	NA	3.53(2.37, 4.78)	12
		**Tolerance**	0.67(0.55, 0.84)	17	0.67(0.58, 0.79)	17	0.68(0.58, 0.81)	17	0.67(0.59, 0.81)	17	0.66(0.54, 0.78)	18	0.67(0.59, 0.81)	11

	**20mg/day**	**Healing**	12.04(7.78, 17.95)	7	11.42(7.94, 17.32)	7	12.45(6.36, 16.97)	7	NA	NA	11.72(4.77, 17.20)	6	11.79(5.93, 17.94)	7
		**Relief of symptoms**	4.09(3.01, 5.39)	9	4.06(3.07, 5.47)	9	NA	NA	NA	NA	NA	NA	3.91(2.90, 5.43)	9
		**Tolerance**	0.64(0.53, 0.76)	11	0.65(0.55, 0.72)	11	0.62(0.53, 0.74)	11	0.64(0.54, 0.72)	11	0.63(0.50, 0.71)	8	0.64(0.57, 0.72)	8
	**40-50mg/day**	**Healing**	15.07(9.58, 23.51)	2	15.24(9.23, 22.41)	2	15.51(8.35, 20.4)	2	NA	NA	14.88(7.78, 22.41)	2	15.32(7.35, 22.95)	2
		**Relief of symptoms**	4.74(3.39, 6.93)	4	4.72(3.45, 7.02)	4	NA	NA	NA	NA	NA	NA	4.47(3.72, 6.82)	4
		**Tolerance**	0.60(0.49, 0.72)	5	0.62(0.52, 0.71)	4	0.63(0.51, 0.72)	7	0.62(0.52, 0.74)	4	0.62(0.50, 0.71)	4	0.62(0.51, 0.72)	4
**Cimetidine**	**200-400mg/day**	**Healing**	NA	NA	NA	NA	NA	NA	NA	NA	NA	NA	NA	NA
		**Relief of symptoms**	1.40(0.92, 1.93)	24	1.38(0.91, 1.92)	24	NA	NA	NA	NA	NA	NA	NA	NA
		**Tolerance**	0.75(0.61, 0.97)	23	0.75(0.62, 0.95)	23	0.77(0.66, 0.97)	23	0.76(0.64, 0.94)	22	0.74(0.65, 0.93)	22	NA	NA
	**600-800mg/day**	**Healing**	NA	NA	NA	NA	NA	NA	NA	NA	NA	NA	NA	NA
		**Relief of symptoms**	1.65(1.17, 2.38)	20	1.60(1.12, 2.32)	20	NA	NA	NA	NA	NA	NA	NA	NA
		**Tolerance**	0.69(0.58, 0.86)	21	0.72(0.60, 0.85)	20	0.72(0.61, 0.86)	21	NA	NA	0.72(0.62, 0.84)	20	NA	NA
	**1600mg/day**	**Healing**	2.87(1.81, 4.28)	19	2.54(1.57, 4.14)	18	2.57(1.83, 4.52)	19	NA	NA	1.57(1.01, 2.72)	18	NA	NA
		**Relief of symptoms**	1.89(1.31, 2.95)	16	1.82(1.31, 3.41)	16	NA	NA	NA	NA	NA	NA	NA	NA
		**Tolerance**	0.65(0.54, 0.81)	13	0.71(0.54, 0.82)	13	0.68(0.60, 0.83)	13	0.67(0.58, 0.79)	13	0.70(0.58, 0.82)	12	NA	NA
**Famotidine**	**20mg/day**	**Healing**	NA	NA	NA	NA	NA	NA	NA	NA	NA	NA	NA	NA
		**Relief of symptoms**	1.46(0.97, 2.00)	23	1.43(0.90, 2.01)	23	NA	NA	NA	NA	NA	NA	1.44(0.97, 2.18)	21
		**Tolerance**	0.72(0.58, 0.94)	22	0.75(0.62, 0.91)	22	0.74(0.63, 0.92)	22	0.72(0.60, 0.90)	21	0.73(0.62, 0.88)	21	0.72(0.62, 0.89)	16
	**40mg/day**	**Healing**	4.15(2.57, 6.42)	16	3.84(2.18, 5.47)	16	4.95(1.78, 6.64)	16	NA	NA	NA	NA	3.97(1.98, 5.78)	16
		**Relief of symptoms**	1.71(1.25, 2.36)	19	1.65(1.20, 2.33)	19	NA	NA	NA	NA	NA	NA	1.69(1.32, 2.24)	18

		**Tolerance**	0.66(0.55, 0.80)	14	0.70(0.60, 0.82)	14	0.71(0.61, 0.80)	14	0.67(0.57, 0.79)	14	0.68(0.56, 0.81)	16	0.69(0.57, 0.75)	13
	**80mg/day**	**Healing**	4.71(2.87, 7.38)	14	4.26(2.65, 6.24)	14	3.92(2.84, 6.21)	14	NA	NA	NA	NA	3.70(2.91, 5.97)	14
		**Relief of symptoms**	1.96(1.38, 2.95)	15	1.91(1.37, 3.00)	15	NA	NA	NA	NA	NA	NA	1.90(1.37, 3.08)	15
		**Tolerance**	NA	NA	NA	NA	NA	NA	NA	NA	NA	NA	NA	NA
**Nizatidine**	**150mg/day**	**Healing**	NA	NA	NA	NA	NA	NA	NA	NA	NA	NA	NA	NA
		**Relief of symptoms**	1.53(1.02, 2.18)	22	1.50(1.04, 2.26)	22	NA	NA	NA	NA	NA	NA	1.52(1.07, 2.13)	20
		**Tolerance**	NA	NA	NA	NA	NA	NA	NA	NA	NA	NA	NA	NA
	**300mg/day**	**Healing**	2.46(1.66, 3.52)	21	2.72(1.65, 3.34)	20	2.75(1.70, 3.32)	21	NA	NA	2.80(1.73, 3.47)	17	2.93(1.71, 3.68)	19
		**Relief of symptoms**	1.82(1.25, 2.82)	17	1.86(1.27, 2.74)	17	NA	NA	NA	NA	NA	NA	1.79(1.23, 2.72)	16
		**Tolerance**	0.69(0.56, 0.87)	20	0.71(0.64, 0.85)	21	0.73(0.63, 0.91)	20	0.72(0.61, 0.87)	20	0.71(0.59, 0.89)	17	0.72(0.59, 0.82)	18
	**600mg/day**	**Healing**	3.01(1.99, 4.44)	18	3.72(1.78, 3.78)	17	3.58(1.79, 3.54)	17	NA	NA	3.03(1.94, 3.66)	14	2.78(1.86, 3.65)	17
		**Relief of symptoms**	NA	NA	NA	NA	NA	NA	NA	NA	NA	NA	NA	NA
		**Tolerance**	0.63(0.51, 0.77)	9	0.65(0.56, 0.79)	9	0.67(0.58, 0.80)	9	0.68(0.57, 0.78)	10	NA	NA	0.68(0.59, 0.75)	9
**Ranitidine**	**≤300mg/day**	**Healing**	2.90(2.06, 3.83)	20	2.77(1.99, 3.82)	21	2.29(1.76, 3.18)	20	NA	NA	2.57(2.26, 2.84)	16	2.19(1.52, 3.48)	20
		**Relief of symptoms**	1.56(1.18, 2.03)	21	1.54(1.17, 2.09)	21	NA	NA	NA	NA	NA	NA	1.54(1.17, 2.14)	19
		**Tolerance**	0.67(0.57, 0.78)	18	0.71(0.54, 0.90)	19	0.68(0.58, 0.80)	19	0.71(0.60, 0.81)	18	0.68(0.61, 0.78)	14	0.68(0.63, 0.76)	17
	**600mg/day**	**Healing**	3.44(2.54, 4.64)	17	3.64(2.73, 4.45)	19	2.99(1.65, 4.76)	18	NA	NA	2.89(2.45, 3.46)	15	2.97(1.56, 3.62)	18
		**Relief of symptoms**	1.75(1.28, 2.47)	18	1.70(1.29, 2.41)	18	NA	NA	NA	NA	NA	NA	1.71(1.22, 2.31)	17
		**Tolerance**	0.61(0.49, 0.73)	8	0.64(0.51, 0.81)	8	0.63(0.53, 0.77)	8	0.61(0.51, 0.72)	8	0.62(0.49, 0.73)	7	0.64(0.50, 0.70)	20
	**1200mg/day**	**Healing**	4.05(2.86, 5.85)	15	3.78(2.92, 5.17)	15	3.97(2.13, 5.32)	15	NA	NA	3.96(2.21, 4.98)	13	3.87(2.34, 5.27)	15

	**Relief of symptoms**	NA	NA	NA	NA	NA	NA	NA	NA	NA	NA	NA	NA
	**Tolerance**	0.58(0.44, 0.71)	4	0.61(0.49, 0.75)	5	0.57(0.51, 0.73)	4	0.61(0.57, 0.73)	7	0.61(0.53, 0.75)	10	0.59(0.54, 0.72)	19
**Placebo**	**Healing**	Ref	22	Ref	22	Ref	22	NA	NA	Ref	19	Ref	21
	**Relief of symptoms**	Ref	25	Ref	25	NA	NA	NA	NA	NA	NA	Ref	22
	**Tolerance**	Ref	24	Ref	24	Ref	24	Ref	23	Ref	23	Ref	21
**Heterogeneity**	**Healing**	0.33(0.22, 0.47)		0.33(0.28, 0.43)		0.34(0.23, 0.42)		NA		0.34(0.16, 0.46)		0.33(0.22, 0.41)	
	**Relief of symptoms**	0.41(0.31, 0.41)*; 0.11(0.004, 0.29)#		0.38(0.30, 0.42)*; 0.11(0.006,0.28)#		NA		NA		NA		0.43(0.33, 0.53)*; 0.11(0.006, 0.28)#	
	**Tolerance**	0.07(0.002, 0.17)		0.06(0.002, 0.13)		0.07(0.003, 0.14)		0.07(0.003,0.14)		0.08(0.005, 0.16)		0.06(0.003, 0.18)	
**Heterogeneity change**	**Healing**	Ref		0%		↑3.0%		NA		↑3.0%		0%	
	**Relief of symptoms**	Ref		↓7.3%; 0%		NA		NA		NA		↑4.9%; 0%	
	**Tolerance**	Ref		↓14.3%		0%		0%		↑14.3%		↓14.3%	

Ref: Reference, NA: Not available, * indicates heterogeneity, # indicates inconsistency.

Supplementary Table S9 Estimates of effects and quality ratings for comparison of recommended doses of different drugs from the main outcome (healing) based on the GRADE system

Comparisons	Direct evidence		Indirect evidence		Network meta-analysis	
	**OR 95%CrIs**	**Quality of evidence**	**OR 95%CrIs**	**Quality of evidence**	**OR 95%CrIs**	**Quality of evidence**
Esomeprazole 20mg *VS.* Placebo	—	—	11.67(7.33, 17.66)	LOW**	11.67(7.33, 17.66)	LOW
Esomeprazole 40mg *VS.* Placebo	—	—	15.45(10.24, 21.96)	LOW**	15.45(10.24, 21.96)	LOW
Lansoprazole 15mg *VS.* Placebo	6.28 (4.33, 9.11)	MODERATE※	12.14(3.13, 26.43)	LOW**	8.23(4.87, 12.79)	LOW‡‡
Lansoprazole 30mg *VS.* Placebo	11.9 (6.78, 21.0)	LOW†,※	12.53(8.18, 19.21)	VERY LOW**	12.30(8.60, 17.03)	LOW
Lansoprazole 60mg *VS.* Placebo	8.78 (3.77, 20.5)	MODERATE※	16.15(10.26, 25.42)	VERY LOW**	14.10(9.44, 21.09)	MODERATE
Omeprazole 20mg *VS.* Placebo	14.3 (9.55, 21.4)	MODERATE†	2.04(0.86, 4.84)	VERY LOW**	10.18(6.93, 14.48)	MODERATE
Omeprazole 40mg *VS.* Placebo	—	—	14.93(9.56, 23.12)	MODERATE¶	14.93(9.56, 23.12)	MODERATE
Pantoprazole 10-20mg *VS.* Placebo	4.35 (2.60, 7.26)	HIGH	67.98(21.58, 214.20)	VERY LOW**	6.88(4.23, 10.82)	HIGH
Pantoprazole 40mg *VS.* Placebo	14.7 (7.71, 28.2)	HIGH	10.63(7.17, 15.76)	LOW**	11.64(8.16, 16.05)	HIGH
Pantoprazole 80mg *VS.* Placebo	—	—	14.74(9.42, 23.30)	LOW**	14.74(9.42, 23.30)	LOW
Rabeprazole 5-10mg *VS.* Placebo	—	—	9.56(5.63, 14.62)	LOW**	9.56(5.63, 14.62)	LOW
Rabeprazole 20mg *VS.* Placebo	—	—	12.04(7.78, 17.95)	MODERATE¶	12.04(7.78, 17.95)	MODERATE
Rabeprazole 40-50mg *VS.* Placebo	—	—	15.07(9.58, 23.51)	LOW**	15.07(9.58, 23.51)	LOW
Cimetidine 1600mg *VS.* Placebo	4.15 (2.13, 8.09)	MODERATE※	2.21(1.26, 3.88)	LOW¶,‡	2.87(1.81, 4.28)	MODERATE
Famotidine 40mg *VS.* Placebo	11.6 (5.90, 22.9)	LOW‡,※	1.76(0.94, 3.26)	VERY LOW**,‡	4.15(2.57, 6.42)	VERY LOW‡‡
Famotidine 80mg *VS.* Placebo	—	—	4.71(2.87, 7.38)	VERY LOW**	4.71(2.87, 7.38)	VERY LOW
Nizatidine 300mg *VS.* Placebo	1.63 (1.01, 2.61)	LOW†,※	4.91(2.65, 9.08)	VERY LOW**	2.46(1.66, 3.52)	LOW
Nizatidine 600mg *VS.* Placebo	1.84 (1.28, 2.63)	MODERATE※	4.16(1.84, 9.40)	VERY LOW**	3.01(1.99, 4.44)	MODERATE
Ranitidine ≤300mg *VS.* Placebo	1.86 (0.96, 3.60)	VERY LOW†,§,※	3.29(2.31, 4.67)	LOW**	2.90(2.06, 3.83)	LOW
Ranitidine 600mg *VS.* Placebo	3.73 (2.05, 6.79)	MODERATE†	3.35(2.36, 4.74)	LOW**	3.44(2.54, 4.64)	MODERATE
Ranitidine 1200mg *VS.* Placebo	4.22 (2.84, 6.27)	HIGH	3.37(1.46, 7.78)	LOW**	4.05(2.86, 5.85)	HIGH

Esomeprazole 40mg *VS.* Esomeprazole 20mg	1.76 (1.17, 2.66)	LOW‡,※	0.84(0.48, 1.45)	VERY LOW**,‡	1.35(1.02, 1.97)	LOW
Lansoprazole 15mg *VS.* Esomeprazole 20mg	—	—	0.73(0.40, 1.14)	VERY LOW**,‡	0.73(0.40, 1.14)	VERY LOW
Lansoprazole 30mg *VS.* Esomeprazole 20mg	—	—	1.08(0.75, 1.58)	VERY LOW**,‡	1.08(0.75, 1.58)	VERY LOW
Lansoprazole 60mg *VS.* Esomeprazole 20mg	—	—	1.24(0.82, 1.90)	VERY LOW**,‡	1.24(0.82, 1.90)	VERY LOW
Omeprazole 20mg *VS.* Esomeprazole 20mg	0.74 (0.59, 0.93)	LOW‡,※	1.55(1.15, 2.08)	LOW**	0.89(0.62, 1.25)	VERY LOW‡
Omeprazole 40mg *VS.* Esomeprazole 20mg	—	—	1.32(0.81, 2.17)	VERY LOW**,‡	1.32(0.81, 2.17)	VERY LOW
Pantoprazole 10-20mg *VS.* Esomeprazole 20mg	—	—	0.61(0.34, 0.998)	VERY LOW**,‡‡	0.61(0.34, 0.998)	VERY LOW
Pantoprazole 40mg *VS.* Esomeprazole 20mg	—	—	1.03(0.70, 1.50)	VERY LOW**,‡	1.03(0.70, 1.50)	VERY LOW
Pantoprazole 80mg *VS.* Esomeprazole 20mg	—	—	1.30(0.82, 2.18)	VERY LOW**,‡	1.30(0.82, 2.18)	VERY LOW
Rabeprazole 5-10mg *VS.* Esomeprazole 20mg	—	—	0.84(0.47, 1.31)	VERY LOW**,‡	0.84(0.47, 1.31)	VERY LOW
Rabeprazole 20mg *VS.* Esomeprazole 20mg	—	—	1.06(0.67, 1.59)	VERY LOW**,‡	1.06(0.67, 1.59)	VERY LOW
Rabeprazole 40-50mg *VS.* Esomeprazole 20mg	—	—	1.32(0.86, 2.07)	VERY LOW**,‡	1.32(0.86, 2.07)	VERY LOW
Cimetidine 1600mg *VS.* Esomeprazole 20mg	—	—	0.26(0.14, 0.42)	LOW**	0.26(0.14, 0.42)	LOW
Famotidine 40mg *VS.* Esomeprazole 20mg	—	—	0.37(0.21, 0.63)	LOW**	0.37(0.21, 0.63)	LOW
Famotidine 80mg *VS.* Esomeprazole 20mg	—	—	0.42(0.23, 0.74)	VERY LOW**	0.42(0.23, 0.74)	VERY LOW
Nizatidine 300mg *VS.* Esomeprazole 20mg	—	—	0.22(0.13, 0.35)	LOW**	0.22(0.13, 0.35)	LOW
Nizatidine 600mg *VS.* Esomeprazole 20mg	—	—	0.27(0.15, 0.44)	LOW**	0.27(0.15, 0.44)	LOW
Ranitidine ≤300mg *VS.* Esomeprazole 20mg	—	—	0.26(0.17, 0.39)	VERY LOW**	0.26(0.17, 0.39)	VERY LOW
Ranitidine 600mg *VS.* Esomeprazole 20mg	—	—	0.31(0.20, 0.46)	LOW**	0.31(0.20, 0.46)	LOW
Ranitidine 1200mg *VS.* Esomeprazole 20mg	—	—	0.36(0.22, 0.58)	LOW**	0.36(0.22, 0.58)	LOW
Lansoprazole 15mg *VS.* Esomeprazole 40mg	—	—	0.54(0.31, 0.85)	MODERATE¶	0.54(0.31, 0.85)	MODERATE
Lansoprazole 30mg *VS.* Esomeprazole 40mg	0.68 (0.58, 0.79)	MODERATE†	1.58(1.32, 1.90)	VERY LOW**	0.81(0.60, 1.06)	LOW‡
Lansoprazole 60mg *VS.* Esomeprazole 40mg	—	—	0.93(0.66, 1.33)	VERY LOW**,‡	0.93(0.66, 1.33)	VERY LOW
Omeprazole 20mg *VS.* Esomeprazole 40mg	0.49 (0.29, 0.84)	LOW†,※	0.76(0.54, 1.06)	VERY LOW**,‡	0.67(0.50, 0.88)	LOW
Omeprazole 40mg *VS.* Esomeprazole 40mg	—	—	0.98(0.65, 1.50)	VERY LOW**,‡	0.98(0.65, 1.50)	VERY LOW
Pantoprazole 10-20mg *VS.* Esomeprazole 40mg	—	—	0.46(0.26, 0.75)	VERY LOW**	0.46(0.26, 0.75)	VERY LOW

Pantoprazole 40mg *VS.* Esomeprazole 40mg	0.91 (0.62, 1.32)	VERY LOW§,※	0.60(0.39, 0.92)	VERY LOW**	0.76(0.57, 1.01)	VERY LOW
Pantoprazole 80mg *VS.* Esomeprazole 40mg	—	—	0.97(0.66, 1.46)	VERY LOW**,‡	0.97(0.66, 1.46)	VERY LOW
Rabeprazole 5-10mg *VS.* Esomeprazole 40mg	—	—	0.63(0.37, 0.92)	LOW**	0.63(0.37, 0.92)	LOW
Rabeprazole 20mg *VS.* Esomeprazole 40mg	—	—	0.79(0.53, 1.09)	VERY LOW**,‡	0.79(0.53, 1.09)	VERY LOW
Rabeprazole 40-50mg *VS.* Esomeprazole 40mg	1.12 (0.80, 1.57)	LOW§	0.34(0.13, 0.88)	LOW**	0.98(0.71, 1.34)	LOW
Cimetidine 1600mg *VS.* Esomeprazole 40mg	—	—	0.19(0.11, 0.30)	LOW**	0.19(0.11, 0.30)	LOW
Famotidine 40mg *VS.* Esomeprazole 40mg	—	—	0.27(0.17, 0.43)	LOW**	0.27(0.17, 0.43)	LOW
Famotidine 80mg *VS.* Esomeprazole 40mg	—	—	0.31(0.19, 0.51)	LOW**	0.31(0.19, 0.51)	LOW
Nizatidine 300mg *VS.* Esomeprazole 40mg	—	—	0.16(0.10, 0.26)	VERY LOW**	0.16(0.10, 0.26)	VERY LOW
Nizatidine 600mg *VS.* Esomeprazole 40mg	—	—	0.20(0.12, 0.32)	VERY LOW**	0.20(0.12, 0.32)	VERY LOW
Ranitidine ≤300mg *VS.* Esomeprazole 40mg	—	—	0.19(0.13, 0.27)	VERY LOW**	0.19(0.13, 0.27)	VERY LOW
Ranitidine 600mg *VS.* Esomeprazole 40mg	—	—	0.23(0.16, 0.33)	MODERATE¶	0.23(0.16, 0.33)	MODERATE
Ranitidine 1200mg *VS.* Esomeprazole 40mg	—	—	0.27(0.18, 0.41)	VERY LOW**	0.27(0.18, 0.41)	VERY LOW
Lansoprazole 30mg *VS.* Lansoprazole 15mg	2.06 (1.41, 3.03)	MODERATE※	0.19(0.09, 0.43)	VERY LOW**	1.56(1.04, 2.49)	MODERATE
Lansoprazole 60mg *VS.* Lansoprazole 15mg	1.56 (0.61, 3.94)	VERY LOW§,※	1.89(1.06, 3.35)	VERY LOW**,‡	1.79(1.13, 3.01)	LOW§§
Omeprazole 20mg *VS.* Lansoprazole 15mg	2.20 (1.45, 3.34)	HIGH	0.01(0.003, 0.03)	VERY LOW**	1.29(0.83, 2.02)	MODERATE‡
Omeprazole 40mg *VS.* Lansoprazole 15mg	—	—	1.91(1.07, 3.39)	MODERATE¶	1.91(1.07, 3.39)	MODERATE
Pantoprazole 10-20mg *VS.* Lansoprazole 15mg	—	—	0.86(0.51, 1.37)	LOW¶,‡	0.86(0.51, 1.37)	LOW
Pantoprazole 40mg *VS.* Lansoprazole 15mg	—	—	1.48(0.93, 2.42)	VERY LOW**,‡	1.48(0.93, 2.42)	VERY LOW
Pantoprazole 80mg *VS.* Lansoprazole 15mg	—	—	1.89(1.07, 3.44)	LOW**	1.89(1.07, 3.44)	LOW
Rabeprazole 5-10mg *VS.* Lansoprazole 15mg	—	—	1.21(0.70, 2.01)	VERY LOW**,‡	1.21(0.70, 2.01)	VERY LOW
Rabeprazole 20mg *VS.* Lansoprazole 15mg	—	—	1.53(0.92, 2.55)	VERY LOW**,‡	1.53(0.92, 2.55)	VERY LOW
Rabeprazole 40-50mg *VS.* Lansoprazole 15mg	—	—	1.92(1.09, 3.43)	LOW**	1.92(1.09, 3.43)	LOW
Cimetidine 1600mg *VS.* Lansoprazole 15mg	—	—	0.37(0.20, 0.63)	MODERATE¶	0.37(0.20, 0.63)	MODERATE
Famotidine 40mg *VS.* Lansoprazole 15mg	—	—	0.54(0.27, 1.01)	LOW**	0.54(0.27, 1.01)	LOW
Famotidine 80mg *VS.* Lansoprazole 15mg	—	—	0.61(0.30, 1.16)	LOW**	0.61(0.30, 1.16)	LOW

Nizatidine 300mg *VS.* Lansoprazole 15mg	—	—	0.31(0.18, 0.53)	LOW**	0.31(0.18, 0.53)	LOW
Nizatidine 600mg *VS.* Lansoprazole 15mg	—	—	0.39(0.22, 0.66)	MODERATE¶	0.39(0.22, 0.66)	MODERATE
Ranitidine ≤300mg *VS.* Lansoprazole 15mg	—	—	0.37(0.22, 0.59)	LOW**	0.37(0.22, 0.59)	LOW
Ranitidine 600mg *VS.* Lansoprazole 15mg	—	—	0.44(0.26, 0.73)	MODERATE¶	0.44(0.26, 0.73)	MODERATE
Ranitidine 1200mg *VS.* Lansoprazole 15mg	—	—	0.52(0.29, 0.93)	MODERATE¶	0.52(0.29, 0.93)	MODERATE
Lansoprazole 60mg *VS.* Lansoprazole 30mg	0.64 (0.21, 1.92)	VERY LOW†,§,※	1.17(0.96, 1.44)	VERY LOW**,‡	1.15(1.003, 1.53)	LOW§§
Omeprazole 20mg *VS.* Lansoprazole 30mg	0.76 (0.53, 1.09)	VERY LOW*, §	0.96(0.63, 1.47)	VERY LOW**,‡	0.84(0.63, 1.09)	LOW
Omeprazole 40mg *VS.* Lansoprazole 30mg	—	—	1.23(0.83, 1.84)	VERY LOW**,‡	1.23(0.83, 1.84)	VERY LOW
Pantoprazole 10-20mg *VS.* Lansoprazole 30mg	—	—	0.57(0.34, 0.88)	VERY LOW**	0.57(0.34, 0.88)	VERY LOW
Pantoprazole 40mg *VS.* Lansoprazole 30mg	0.71 (0.34, 1.48)	VERY LOW*, §	1.02(0.73, 1.42)	VERY LOW**,‡	0.96(0.70, 1.28)	VERY LOW
Pantoprazole 80mg *VS.* Lansoprazole 30mg	—	—	1.21(0.82, 1.89)	VERY LOW**,‡	1.21(0.82, 1.89)	VERY LOW
Rabeprazole 5-10mg *VS.* Lansoprazole 30mg	—	—	0.78(0.47, 1.14)	VERY LOW**,‡	0.78(0.47, 1.14)	VERY LOW
Rabeprazole 20mg *VS.* Lansoprazole 30mg	0.88 (0.32, 2.40)	VERY LOW*, §	1.01(0.68, 1.51)	VERY LOW**,‡	0.99(0.66, 1.39)	VERY LOW
Rabeprazole 40-50mg *VS.* Lansoprazole 30mg	—	—	1.24(0.84, 1.82)	VERY LOW**,‡,‡‡	1.24(0.84, 1.82)	VERY LOW
Cimetidine 1600mg *VS.* Lansoprazole 30mg	—	—	0.24(0.14, 0.37)	LOW**	0.24(0.14, 0.37)	LOW
Famotidine 40mg *VS.* Lansoprazole 30mg	—	—	0.34(0.21, 0.54)	LOW**	0.34(0.21, 0.54)	LOW
Famotidine 80mg *VS.* Lansoprazole 30mg	—	—	0.39(0.23, 0.64)	LOW**	0.39(0.23, 0.64)	LOW
Nizatidine 300mg *VS.* Lansoprazole 30mg	—	—	0.20(0.12, 0.31)	LOW**	0.20(0.12, 0.31)	LOW
Nizatidine 600mg *VS.* Lansoprazole 30mg	—	—	0.25(0.15, 0.39)	LOW**	0.25(0.15, 0.39)	LOW
Ranitidine ≤300mg *VS.* Lansoprazole 30mg	0.16 (0.06, 0.40)	LOW†,※	0.25(0.18, 0.35)	VERY LOW**	0.24(0.17, 0.32)	LOW
Ranitidine 600mg *VS.* Lansoprazole 30mg	0.19 (0.07, 0.51)	MODERATE※	0.31(0.21, 0.45)	LOW**	0.29(0.20, 0.40)	MODERATE
Ranitidine 1200mg *VS.* Lansoprazole 30mg	—	—	0.34(0.23, 0.51)	LOW**	0.34(0.23, 0.51)	LOW
Omeprazole 20mg *VS.* Lansoprazole 60mg	—	—	0.74(0.49, 1.001)	VERY LOW**	0.74(0.49, 1.001)	VERY LOW
Omeprazole 40mg *VS.* Lansoprazole 60mg	—	—	1.08(0.68, 1.65)	LOW¶,‡	1.08(0.68, 1.65)	LOW
Pantoprazole 10-20mg *VS.* Lansoprazole 60mg	—	—	0.50(0.28, 0.80)	LOW¶,‡‡	0.50(0.28, 0.80)	LOW
Pantoprazole 40mg *VS.* Lansoprazole 60mg	—	—	0.84(0.57, 1.17)	VERY LOW**,‡	0.84(0.57, 1.17)	VERY LOW

Pantoprazole 80mg *VS.* Lansoprazole 60mg	—	—	1.07(0.68, 1.69)	VERY LOW**,‡	1.07(0.68, 1.69)	VERY LOW
Rabeprazole 5-10mg *VS.* Lansoprazole 60mg	—	—	0.69(0.39, 1.02)	VERY LOW**,‡	0.69(0.39, 1.02)	VERY LOW
Rabeprazole 20mg *VS.* Lansoprazole 60mg	—	—	0.87(0.55, 1.25)	VERY LOW**,‡	0.87(0.55, 1.25)	VERY LOW
Rabeprazole 40-50mg *VS.* Lansoprazole 60mg	—	—	1.09(0.69, 1.62)	VERY LOW**,‡	1.09(0.69, 1.62)	VERY LOW
Cimetidine 1600mg *VS.* Lansoprazole 60mg	—	—	0.21(0.12, 0.34)	VERY LOW**	0.21(0.12, 0.34)	VERY LOW
Famotidine 40mg *VS.* Lansoprazole 60mg	—	—	0.30(0.18, 0.49)	LOW**	0.30(0.18, 0.49)	LOW
Famotidine 80mg *VS.* Lansoprazole 60mg	—	—	0.34(0.20, 0.56)	LOW**	0.34(0.20, 0.56)	LOW
Nizatidine 300mg *VS.* Lansoprazole 60mg	—	—	0.18(0.10, 0.28)	LOW**	0.18(0.10, 0.28)	LOW
Nizatidine 600mg *VS.* Lansoprazole 60mg	—	—	0.22(0.13, 0.35)	MODERATE¶	0.22(0.13, 0.35)	MODERATE
Ranitidine ≤300mg *VS.* Lansoprazole 60mg	0.25 (0.12, 0.51)	MODERATE※	0.20(0.13, 0.31)	VERY LOW**	0.21(0.14, 0.30)	MODERATE
Ranitidine 600mg *VS.* Lansoprazole 60mg	—	—	0.25(0.17, 0.36)	MODERATE¶	0.25(0.17, 0.36)	MODERATE
Ranitidine 1200mg *VS.* Lansoprazole 60mg	—	—	0.30(0.19, 0.46)	MODERATE¶	0.30(0.19, 0.46)	MODERATE
Omeprazole 40mg *VS.* Omeprazole 20mg	—	—	1.49(1.03, 2.31)	VERY LOW**	1.49(1.03, 2.31)	VERY LOW
Pantoprazole 10-20mg *VS.* Omeprazole 20mg	0.56 (0.30, 1.04)	VERY LOW*, §	0.83(0.44, 1.57)	VERY LOW**,‡	0.68(0.42, 1.02)	LOW§§
Pantoprazole 40mg *VS.* Omeprazole 20mg	1.14 (0.56, 2.35)	VERY LOW*,†,§	1.16(0.84, 1.62)	VERY LOW**,‡	1.16(0.85, 1.55)	VERY LOW
Pantoprazole 80mg *VS.* Omeprazole 20mg	—	—	1.47(0.99, 2.29)	VERY LOW**,‡	1.47(0.99, 2.29)	VERY LOW
Rabeprazole 5-10mg *VS.* Omeprazole 20mg	0.65 (0.22, 1.89)	LOW§	1.02(0.64, 1.64)	VERY LOW**,‡	0.95(0.58, 1.38)	LOW
Rabeprazole 20mg *VS.* Omeprazole 20mg	1.36 (0.35, 5.28)	VERY LOW*,†,§,※	1.19(0.81, 1.74)	VERY LOW**,‡	1.20(0.82, 1.70)	VERY LOW
Rabeprazole 40-50mg *VS.* Omeprazole 20mg	0.65 (0.22, 1.91)	LOW§	1.69(1.12, 2.53)	VERY LOW**,‡‡	1.50(1.02, 2.18)	MODERATE§§
Cimetidine 1600mg *VS.* Omeprazole 20mg	—	—	0.29(0.17, 0.45)	MODERATE¶	0.29(0.17, 0.45)	MODERATE
Famotidine 40mg *VS.* Omeprazole 20mg	0.29(0.16, 0.51)	MODERATE※	1.11(0.43, 2.82)	LOW**	0.42(0.25, 0.67)	MODERATE
Famotidine 80mg *VS.* Omeprazole 20mg	—	—	0.47(0.27, 0.80)	VERY LOW**	0.47(0.27, 0.80)	VERY LOW
Nizatidine 300mg *VS.* Omeprazole 20mg	—	—	0.25(0.15, 0.37)	VERY LOW**	0.25(0.15, 0.37)	VERY LOW
Nizatidine 600mg *VS.* Omeprazole 20mg	—	—	0.30(0.19, 0.47)	MODERATE¶	0.30(0.19, 0.47)	MODERATE
Ranitidine ≤300mg *VS.* Omeprazole 20mg	—	—	0.29(0.20, 0.40)	VERY LOW**	0.29(0.20, 0.40)	VERY LOW
Ranitidine 600mg *VS.* Omeprazole 20mg	—	—	0.35(0.24, 0.49)	VERY LOW**	0.35(0.24, 0.49)	VERY LOW

Ranitidine 1200mg *VS.* Omeprazole 20mg	—	—	0.41(0.27, 0.63)	MODERATE¶	0.41(0.27, 0.63)	MODERATE
Pantoprazole 10-20mg *VS.* Omeprazole 40mg	—	—	0.48(0.25, 0.81)	MODERATE¶	0.48(0.25, 0.81)	MODERATE
Pantoprazole 40mg *VS.* Omeprazole 40mg	0.96 (0.70, 1.32)	LOW§	0.72(0.42, 1.25)	LOW¶,‡	0.80(0.52, 1.13)	LOW
Pantoprazole 80mg *VS.* Omeprazole 40mg	—	—	1.02(0.62, 1.59)	VERY LOW**,‡	1.02(0.62, 1.59)	VERY LOW
Rabeprazole 5-10mg *VS.* Omeprazole 40mg	—	—	0.66(0.35, 1.03)	VERY LOW**,‡	0.66(0.35, 1.03)	VERY LOW
Rabeprazole 20mg *VS.* Omeprazole 40mg	—	—	0.83(0.49, 1.24)	VERY LOW**,‡	0.83(0.49, 1.24)	VERY LOW
Rabeprazole 40-50mg *VS.* Omeprazole 40mg	—	—	1.04(0.64, 1.57)	VERY LOW**,‡	1.04(0.64, 1.57)	VERY LOW
Cimetidine 1600mg *VS.* Omeprazole 40mg	0.12 (0.04, 0.38)	HIGH	0.23(0.13, 0.43)	MODERATE¶	0.20(0.11, 0.32)	HIGH
Famotidine 40mg *VS.* Omeprazole 40mg	—	—	0.29(0.16, 0.47)	LOW**	0.29(0.16, 0.47)	LOW
Famotidine 80mg *VS.* Omeprazole 40mg	—	—	0.33(0.19, 0.55)	LOW**	0.33(0.19, 0.55)	LOW
Nizatidine 300mg *VS.* Omeprazole 40mg	—	—	0.17(0.09, 0.28)	LOW**	0.17(0.09, 0.28)	LOW
Nizatidine 600mg *VS.* Omeprazole 40mg	—	—	0.21(0.12, 0.35)	MODERATE¶	0.21(0.12, 0.35)	MODERATE
Ranitidine ≤300mg *VS.* Omeprazole 40mg	0.20 (0.11, 0.38)	HIGH	0.20(0.12, 0.34)	LOW**	0.20(0.13, 0.29)	HIGH
Ranitidine 600mg *VS.* Omeprazole 40mg	0.10 (0.04, 0.27)	HIGH	0.30(0.19, 0.48)	LOW**	0.24(0.15, 0.35)	HIGH
Ranitidine 1200mg *VS.* Omeprazole 40mg	—	—	0.28(0.17, 0.43)	LOW**	0.28(0.17, 0.43)	LOW
Pantoprazole 40mg *VS.* Pantoprazole 10-20mg	3.24 (1.99, 5.28)	HIGH	0.03(0.01, 0.11)	LOW†	1.77(1.09, 2.71)	HIGH
Pantoprazole 80mg *VS.* Pantoprazole 10-20mg	—	—	2.25(1.25, 3.96)	LOW**	2.25(1.25, 3.96)	LOW
Rabeprazole 5-10mg *VS.* Pantoprazole 10-20mg	—	—	1.44(0.83, 2.34)	VERY LOW**,‡,‡‡	1.44(0.83, 2.34)	VERY LOW
Rabeprazole 20mg *VS.* Pantoprazole 10-20mg	—	—	1.83(1.07, 3.02)	VERY LOW**,‡‡	1.83(1.07, 3.02)	VERY LOW
Rabeprazole 40-50mg *VS.* Pantoprazole 10-20mg	—	—	2.30(1.23, 3.99)	VERY LOW**,‡‡	2.30(1.23, 3.99)	VERY LOW
Cimetidine 1600mg *VS.* Pantoprazole 10-20mg	—	—	0.44(0.24, 0.72)	VERY LOW**,‡‡	0.44(0.24, 0.72)	VERY LOW
Famotidine 40mg *VS.* Pantoprazole 10-20mg	—	—	0.64(0.31, 1.17)	VERY LOW**	0.64(0.31, 1.17)	VERY LOW
Famotidine 80mg *VS.* Pantoprazole 10-20mg	—	—	0.73(0.34, 1.35)	VERY LOW**,‡,‡‡	0.73(0.34, 1.35)	VERY LOW
Nizatidine 300mg *VS.* Pantoprazole 10-20mg	—	—	0.37(0.22, 0.60)	MODERATE¶	0.37(0.22, 0.60)	MODERATE
Nizatidine 600mg *VS.* Pantoprazole 10-20mg	—	—	0.46(0.26, 0.76)	MODERATE¶	0.46(0.26, 0.76)	MODERATE
Ranitidine ≤300mg *VS.* Pantoprazole 10-20mg	0.49 (0.32, 0.77)	MODERATE※	0.42(0.08, 2.47)	MODERATE¶	0.44(0.27, 0.67)	MODERATE

Ranitidine 600mg *VS.* Pantoprazole 10-20mg	—	—	0.53(0.31, 0.84)	VERY LOW**,‡‡	0.53(0.31, 0.84)	VERY LOW
Ranitidine 1200mg *VS.* Pantoprazole 10-20mg	—	—	0.62(0.34, 1.07)	HIGH∏	0.62(0.34, 1.07)	HIGH
Pantoprazole 80mg *VS.* Pantoprazole 40mg	1.21 (0.68, 2.16)	LOW §	1.30(0.89, 1.88)	LOW¶,‡	1.27(1.01, 1.89)	MODERATE §§
Rabeprazole 5-10mg *VS.* Pantoprazole 40mg	—	—	0.83(0.50, 1.21)	VERY LOW**,‡	0.83(0.50, 1.21)	VERY LOW
Rabeprazole 20mg *VS.* Pantoprazole 40mg	1.39 (0.55, 3.51)	VERY LOW*, §	0.99(0.66, 1.47)	VERY LOW**,‡	1.04(0.71, 1.48)	VERY LOW
Rabeprazole 40-50mg *VS.* Pantoprazole 40mg	—	—	1.30(0.89, 1.90)	VERY LOW**,‡,‡‡	1.30(0.89, 1.90)	VERY LOW
Cimetidine 1600mg *VS.* Pantoprazole 40mg	—	—	0.25(0.15, 0.39)	VERY LOW**	0.25(0.15, 0.39)	VERY LOW
Famotidine 40mg *VS.* Pantoprazole 40mg	—	—	0.36(0.22, 0.57)	LOW**	0.36(0.22, 0.57)	LOW
Famotidine 80mg *VS.* Pantoprazole 40mg	—	—	0.41(0.25, 0.66)	LOW**	0.41(0.25, 0.66)	LOW
Nizatidine 300mg *VS.* Pantoprazole 40mg	0.26 (0.14, 0.47)	MODERATE†	0.19(0.11, 0.33)	LOW**	0.22(0.14, 0.32)	MODERATE
Nizatidine 600mg *VS.* Pantoprazole 40mg	—	—	0.26(0.16, 0.41)	VERY LOW**	0.26(0.16, 0.41)	VERY LOW
Ranitidine ≤300mg *VS.* Pantoprazole 40mg	0.32 (0.16, 0.62)	MODERATE†	0.24(0.17, 0.33)	LOW¶,‡‡	0.25(0.19, 0.34)	MODERATE
Ranitidine 600mg *VS.* Pantoprazole 40mg	—	—	0.30(0.21, 0.42)	MODERATE ¶	0.30(0.21, 0.42)	MODERATE
Ranitidine 1200mg *VS.* Pantoprazole 40mg	—	—	0.35(0.24, 0.53)	HIGH∏	0.35(0.24, 0.53)	HIGH
Rabeprazole 5-10mg *VS.* Pantoprazole 80mg	—	—	0.67(0.35, 1.05)	VERY LOW**,‡	0.67(0.35, 1.05)	VERY LOW
Rabeprazole 20mg *VS.* Pantoprazole 80mg	—	—	0.84(0.50, 1.26)	VERY LOW**,‡	0.84(0.50, 1.26)	VERY LOW
Rabeprazole 40-50mg *VS.* Pantoprazole 80mg	—	—	1.05(0.63, 1.61)	VERY LOW**,‡	1.05(0.63, 1.61)	VERY LOW
Cimetidine 1600mg *VS.* Pantoprazole 80mg	—	—	0.20(0.11, 0.33)	LOW**	0.20(0.11, 0.33)	LOW
Famotidine 40mg *VS.* Pantoprazole 80mg	—	—	0.29(0.17, 0.47)	LOW**	0.29(0.17, 0.47)	LOW
Famotidine 80mg *VS.* Pantoprazole 80mg	—	—	0.33(0.18, 0.56)	LOW**	0.33(0.18, 0.56)	LOW
Nizatidine 300mg *VS.* Pantoprazole 80mg	0.15 (0.07, 0.32)	HIGH	0.19(0.09, 0.38)	LOW**	0.17(0.10, 0.28)	HIGH
Nizatidine 600mg *VS.* Pantoprazole 80mg	—	—	0.21(0.12, 0.35)	VERY LOW**	0.21(0.12, 0.35)	VERY LOW
Ranitidine ≤300mg *VS.* Pantoprazole 80mg	—	—	0.20(0.12, 0.29)	LOW**	0.20(0.12, 0.29)	LOW
Ranitidine 600mg *VS.* Pantoprazole 80mg	—	—	0.24(0.15, 0.36)	LOW**	0.24(0.15, 0.36)	LOW
Ranitidine 1200mg *VS.* Pantoprazole 80mg	—	—	0.29(0.17, 0.45)	LOW**	0.29(0.17, 0.45)	LOW
Rabeprazole 20mg *VS.* Rabeprazole 5-10mg	—	—	1.29(1.01, 2.01)	VERY LOW**	1.29(1.01, 2.01)	VERY LOW

Rabeprazole 40-50mg *VS.* Rabeprazole 5-10mg	1.01 (0.38, 2.66)	LOW§	1.87(1.11, 3.15)	LOW**	1.63(1.09, 2.73)	LOW
Cimetidine 1600mg *VS.* Rabeprazole 5-10mg	—	—	0.32(0.17, 0.54)	LOW**	0.32(0.17, 0.54)	LOW
Famotidine 40mg *VS.* Rabeprazole 5-10mg	—	—	0.46(0.24, 0.86)	LOW**	0.46(0.24, 0.86)	LOW
Famotidine 80mg *VS.* Rabeprazole 5-10mg	—	—	0.52(0.27, 0.98)	VERY LOW**	0.52(0.27, 0.98)	VERY LOW
Nizatidine 300mg *VS.* Rabeprazole 5-10mg	—	—	0.27(0.16, 0.45)	LOW**	0.27(0.16, 0.45)	LOW
Nizatidine 600mg *VS.* Rabeprazole 5-10mg	—	—	0.33(0.19, 0.58)	LOW**	0.33(0.19, 0.58)	LOW
Ranitidine ≤300mg *VS.* Rabeprazole 5-10mg	—	—	0.32(0.20, 0.50)	VERY LOW**	0.32(0.20, 0.50)	VERY LOW
Ranitidine 600mg *VS.* Rabeprazole 5-10mg	—	—	0.38(0.24, 0.63)	VERY LOW**	0.38(0.24, 0.63)	VERY LOW
Ranitidine 1200mg *VS.* Rabeprazole 5-10mg	—	—	0.45(0.26, 0.78)	LOW**	0.45(0.26, 0.78)	LOW
Rabeprazole 40-50mg *VS.* Rabeprazole 20mg	—	—	1.27(1.01, 1.89)	VERY LOW**	1.27(1.01, 1.89)	VERY LOW
Cimetidine 1600mg *VS.* Rabeprazole 20mg	—	—	0.25(0.14, 0.40)	MODERATE¶	0.25(0.14, 0.40)	MODERATE
Famotidine 40mg *VS.* Rabeprazole 20mg	—	—	0.36(0.21, 0.61)	LOW**	0.36(0.21, 0.61)	LOW
Famotidine 80mg *VS.* Rabeprazole 20mg	—	—	0.40(0.23, 0.69)	LOW**	0.40(0.23, 0.69)	LOW
Nizatidine 300mg *VS.* Rabeprazole 20mg	—	—	0.21(0.12, 0.34)	VERY LOW**	0.21(0.12, 0.34)	VERY LOW
Nizatidine 600mg *VS.* Rabeprazole 20mg	—	—	0.26(0.15, 0.43)	VERY LOW**	0.26(0.15, 0.43)	VERY LOW
Ranitidine ≤300mg *VS.* Rabeprazole 20mg	—	—	0.25(0.16, 0.36)	LOW**	0.25(0.16, 0.36)	LOW
Ranitidine 600mg *VS.* Rabeprazole 20mg	0.28 (0.16, 0.49)	MODERATE*	0.33(0.17, 0.62)	VERY LOW**	0.3(0.19, 0.44)	MODERATE
Ranitidine 1200mg *VS.* Rabeprazole 20mg	—	—	0.35(0.22, 0.56)	LOW**	0.35(0.22, 0.56)	LOW
Cimetidine 1600mg *VS.* Rabeprazole 40-50mg	—	—	0.20(0.10, 0.33)	LOW**	0.20(0.10, 0.33)	LOW
Famotidine 40mg *VS.* Rabeprazole 40-50mg	—	—	0.29(0.17, 0.47)	LOW**	0.29(0.17, 0.47)	LOW
Famotidine 80mg *VS.* Rabeprazole 40-50mg	—	—	0.32(0.19, 0.54)	LOW**	0.32(0.19, 0.54)	LOW
Nizatidine 300mg *VS.* Rabeprazole 40-50mg	—	—	0.17(0.09, 0.28)	LOW**	0.17(0.09, 0.28)	LOW
Nizatidine 600mg *VS.* Rabeprazole 40-50mg	—	—	0.21(0.11, 0.34)	LOW**	0.21(0.11, 0.34)	LOW
Ranitidine ≤300mg *VS.* Rabeprazole 40-50mg	—	—	0.20(0.12, 0.30)	LOW**	0.20(0.12, 0.30)	LOW
Ranitidine 600mg *VS.* Rabeprazole 40-50mg	—	—	0.24(0.15, 0.36)	LOW**	0.24(0.15, 0.36)	LOW
Ranitidine 1200mg *VS.* Rabeprazole 40-50mg	—	—	0.28(0.17, 0.45)	LOW**	0.28(0.17, 0.45)	LOW

Famotidine 40mg *VS.* Cimetidine 1600mg	—	—	1.50(0.86, 2.62)	VERY LOW**,‡	1.50(0.86, 2.62)	VERY LOW
Famotidine 80mg *VS.* Cimetidine 1600mg	—	—	1.70(0.96, 2.99)	VERY LOW**,‡	1.70(0.96, 2.99)	VERY LOW
Nizatidine 300mg *VS.* Cimetidine 1600mg	—	—	0.89(0.52, 1.40)	VERY LOW**,‡	0.89(0.52, 1.40)	VERY LOW
Nizatidine 600mg *VS.* Cimetidine 1600mg	—	—	1.09(0.63, 1.79)	LOW¶,‡	1.09(0.63, 1.79)	LOW
Ranitidine ≤300mg *VS.* Cimetidine 1600mg	1.17 (0.82, 1.69)	VERY LOW*, §	0.62(0.31, 1.28)	MODERATE∏,‡	1.05(0.67, 1.55)	MODERATE
Ranitidine 600mg *VS.* Cimetidine 1600mg	1.86 (1.28, 2.72)	MODERATE*	0.08(0.03, 0.20)	MODERATE ¶	1.24(0.81, 1.86)	LOW‡
Ranitidine 1200mg *VS.* Cimetidine 1600mg	—	—	1.46(0.92, 2.34)	LOW¶,‡	1.46(0.92, 2.34)	LOW
Famotidine 80mg *VS.* Famotidine 40mg	0.81 (0.49, 1.34)	LOW §	1.22(0.98, 1.51)	VERY LOW**,‡	1.14(1.004, 1.51)	MODERATE §§
Nizatidine 300mg *VS.* Famotidine 40mg	—	—	0.62(0.33, 1.01)	VERY LOW**,‡	0.62(0.33, 1.01)	VERY LOW
Nizatidine 600mg *VS.* Famotidine 40mg	—	—	0.76(0.40, 1.23)	VERY LOW**,‡	0.76(0.40, 1.23)	VERY LOW
Ranitidine ≤300mg *VS.* Famotidine 40mg	0.74 (0.45, 1.23)	LOW §	0.70(0.31, 1.60)	VERY LOW**,‡	0.73(0.45, 1.06)	LOW
Ranitidine 600mg *VS.* Famotidine 40mg	—	—	0.86(0.54, 1.26)	VERY LOW**,‡	0.86(0.54, 1.26)	VERY LOW
Ranitidine 1200mg *VS.* Famotidine 40mg	—	—	1.01(0.62, 1.55)	VERY LOW**,‡	1.01(0.62, 1.55)	VERY LOW
Nizatidine 300mg *VS.* Famotidine 80mg	—	—	0.55(0.28, 0.89)	LOW**	0.55(0.28, 0.89)	LOW
Nizatidine 600mg *VS.* Famotidine 80mg	—	—	0.67(0.35, 1.11)	VERY LOW**,‡	0.67(0.35, 1.11)	VERY LOW
Ranitidine ≤300mg *VS.* Famotidine 80mg	0.92 (0.60, 1.40)	LOW §	0.03(0.01, 0.13)	LOW**	0.64(0.39, 0.95)	LOW
Ranitidine 600mg *VS.* Famotidine 80mg	—	—	0.76(0.46, 1.11)	VERY LOW**,‡	0.76(0.46, 1.11)	VERY LOW
Ranitidine 1200mg *VS.* Famotidine 80mg	—	—	0.90(0.54, 1.36)	VERY LOW**,‡	0.90(0.54, 1.36)	VERY LOW
Nizatidine 600mg *VS.* Nizatidine 300mg	1.15 (0.67, 1.97)	VERY LOW†,§,※	1.28(0.92, 1.77)	VERY LOW**,‡	1.24(1.01, 1.77)	LOW §§
Ranitidine ≤300mg *VS.* Nizatidine 300mg	—	—	1.21(0.81, 1.81)	LOW¶,‡	1.21(0.81, 1.81)	LOW
Ranitidine 600mg *VS.* Nizatidine 300mg	—	—	1.44(0.96, 2.16)	VERY LOW**,‡	1.44(0.96, 2.16)	VERY LOW
Ranitidine 1200mg *VS.* Nizatidine 300mg	—	—	1.70(1.08, 2.67)	VERY LOW**,‡	1.70(1.08, 2.67)	VERY LOW
Ranitidine ≤300mg *VS.* Nizatidine 600mg	—	—	0.99(0.64, 1.47)	VERY LOW**,‡	0.99(0.64, 1.47)	VERY LOW
Ranitidine 600mg *VS.* Nizatidine 600mg	—	—	1.18(0.75, 1.77)	LOW¶,‡	1.18(0.75, 1.77)	LOW
Ranitidine 1200mg *VS.* Nizatidine 600mg	—	—	1.39(0.86, 2.21)	LOW¶,‡	1.39(0.86, 2.21)	LOW
Ranitidine 600mg *VS.* Ranitidine ≤300mg	1.59 (1.09, 2.31)	LOW*,‡	1.01(0.76, 1.36)	VERY LOW**	1.20(1.01, 1.60)	LOW

Ranitidine 1200mg *VS*. Ranitidine ≤300mg	—	—	1.41(1.06, 2.07)	VERY LOW**	1.41(1.06, 2.07)	VERY LOW
Ranitidine 1200mg *VS*. Ranitidine 600mg	0.84 (0.57, 1.24)	LOW§	1.44(1.07, 1.94)	LOW¶,‡	1.18(1.01, 1.62)	MODERATE§§

—Not applicable. *Limitations (risk of bias). †Inconsistency. ‡Imprecision. §Severe imprecision. ※Publication bias. **Contributing direct evidence of low or very low quality. ¶Contributing direct evidence of moderate quality. ∏Contributing direct evidence of high quality. ‡‡Indirectness because of questionable comparability of trial populations to target population of NMA (level of disease from endoscopy) or because of intransitivity. §§Greater precision.

Supplementary Figure Legends

Supplementary Figure S1a. Risk of bias summary (continued)

Supplementary Figure S1b. Risk of bias summary (continued)

Supplementary Figure S1c. Risk of bias summary (continued)

Supplementary Figure S1d. Risk of bias summary

Supplementary Figure S1a. Risk of bias summary (continued)

	Random sequence generation (selection bias)	Allocation concealment (selection bias)	Blinding of participants and personnel (performance bias)	Blinding of outcome assessment (detection bias)	Incomplete outcome data (attrition bias)	Selective reporting (reporting bias)	Other bias
Adachi 2003	?	+	?	?	?	+	?
Armstrong 2001	+	?	+	?	+	+	+
Armstrong a 2004	?	?	?	?	+	+	+
Armstrong b 2004	?	?	?	?	+	+	+
Armstrong c 2004	?	?	?	?	+	+	+
Bardhan 1995	+	+	+	+	+	+	+
Bardhan 2001	+	?	−	−	+	+	+
Bardhan 2007	+	+	+	?	+	+	+
Bate 1996	?	?	+	?	+	+	?
Bate 1997	?	?	?	?	+	+	?
Bytzer 2006	+	+	+	?	+	+	?
Castell 1996	?	?	+	?	+	+	?
Castell 2002	+	+	+	?	+	+	+
Cho 2012	+	?	+	?	+	+	?
Cloud 1992	+	?	+	?	+	+	+
Dehn 1990	?	?	+	?	?	+	?
Delchier 2000	?	?	+	?	+	+	?
Dettmer 1998	?	?	+	?	+	+	+
Earnest 1998	?	?	+	?	+	+	?
Eggleston 2009	?	?	+	+	+	+	+
Euler 1993	?	?	+	?	?	+	?
Farley 2000	?	?	+	+	+	+	+
Fass 2000	+	?	−	−	+	+	?
Fennerty 2005	+	+	+	+	+	+	?
Festen 1999	+	?	+	?	+	+	+

Supplementary Figure S1b. Risk of bias summary (continued)

	Random sequence generation (selection bias)	Allocation concealment (selection bias)	Blinding of participants and personnel (performance bias)	Blinding of outcome assessment (detection bias)	Incomplete outcome data (attrition bias)	Selective reporting (reporting bias)	Other bias
Fock 2005	+	?	+	+	+	+	?
Galmiche 1997	?	?	+	?	+	+	?
Galmiche 1998	?	?	+	?	+	+	+
Gillessen 2004	+	?	+	?	+	+	+
Glatzel 2007	?	?	+	+	+	+	+
Havelund 1988	+	+	+	?	+	+	+
Holtmann 2002	+	?	+	+	+	+	+
Hongo 2008	+	+	+	?	+	+	+
Jansen 1999	?	+	+	?	+	+	+
Johnson 2005	+	+	+	+	+	+	+
Johnson 2010	+	+	+	+	+	+	+
Jones 1999	+	?	+	?	+	+	?
Kahrilas 2000	?	+	+	+	+	+	+
Kahrilas 2005	?	?	+	?	+	+	+
Kao 2003	?	?	+	+	+	+	?
Kaspari 2001	?	?	+	+	+	+	?
Katz a 2003	+	+	+	+	+	+	+
Katz b 2003	+	+	+	+	+	+	+
Kim 2015	+	?	+	−	+	+	?
Kinoshita 2011	+	+	+	+	+	+	?
Kobeissy 2012	+	?	−	−	+	+	+
Koop 1995	?	?	+	+	+	+	?
Korner 2003	+	?	+	+	+	+	+
Kovacs 2002	?	?	+	?	+	+	?
Kushner a 2009	?	?	+	+	+	+	+
Kushner b 2009	?	?	+	+	+	+	+

Supplementary Figure S1c. Risk of bias summary (continued)

	Random sequence generation (selection bias)	Allocation concealment (selection bias)	Blinding of participants and personnel (performance bias)	Blinding of outcome assessment (detection bias)	Incomplete outcome data (attrition bias)	Selective reporting (reporting bias)	Other bias
Laine a 2011	+	+	+	+	+	+	+
Laine b 2011	+	+	+	+	+	+	+
Lightdale 2006	+	+	+	+	+	+	?
Lundell 1990	?	?	+	?	+	+	+
Maton 1999	?	?	+	?	+	+	+
Mccarty Dawson 1996	?	?	−	?	+	+	?
Mee 1996	+	+	+	+	+	+	+
Meneghelli 2002	+	?	+	+	+	+	+
Miner 2002	?	?	+	?	+	+	?
Monnikes 2007	+	+	+	?	+	+	+
Moraes Filho 2014	+	+	+	+	+	+	?
Mossner 1995	+	?	+	?	+	+	+
Mulder 2002	?	?	+	?	+	+	+
Ohara 2010	+	+	+	?	+	+	+
Pace 2005	+	?	−	−	+	+	?
Palmer 1990	?	?	+	?	+	+	+
Pappa a 1999	?	?	+	?	+	+	+
Pappa b 1999	?	?	+	?	+	+	?
Paul 2001	+	?	+	?	+	+	?
Peura 2009	?	?	+	?	+	+	?
Peura a 2014	+	+	+	?	+	+	?
Peura b 2014	+	+	+	?	+	+	?
Pilotto 2007	+	?	−	−	+	+	?
Quik 1990	?	?	+	?	+	+	?
Richer 1 2000	?	?	+	?	+	+	?

Supplementary Figure S1d. Risk of bias summary

	Random sequence generation (selection bias)	Allocation concealment (selection bias)	Blinding of participants and personnel (performance bias)	Blinding of outcome assessment (detection bias)	Incomplete outcome data (attrition bias)	Selective reporting (reporting bias)	Other bias
Richer 2 2000	?	?	+	?	+	+	?
Richter 1 2001	?	?	+	+	+	+	?
Richter 1999	?	?	+	?	+	+	?
Richter 2 2001	+	+	+	+	+	+	+
Riemann 1991	?	?	+	?	?	?	?
Robinson 1991	?	?	?	?	+	+	?
Robinson 1995	?	?	+	?	+	+	?
Robinson 1996	+	?	+	+	+	+	+
Roufail 1992	?	?	+	?	+	+	?
Sabesin 1991	?	?	+	?	+	+	?
Schmitt 2006	+	+	+	+	+	+	?
Scholten 2003	+	?	+	?	+	+	?
Silver 1996	?	?	+	?	+	+	?
Simon 1993	?	?	+	?	+	+	?
Sontag 1987	?	?	+	?	+	+	+
Tan 2011	+	+	+	+	+	+	+
Uemura 2008	?	?	+	?	+	+	?
Vantrappen 1988	?	?	+	?	+	+	+
Van Zyl 2000	+	?	+	+	+	+	+
Van Zyl 2004	+	?	+	?	+	+	+
Venables 1997	?	?	+	?	+	+	+
Zheng 2009	?	+	?	?	+	+	?
